# New Vistas in microRNA Regulatory Interactome in Neuropathic Pain

**DOI:** 10.3389/fphar.2021.778014

**Published:** 2022-02-25

**Authors:** Yash Gada, Amitkumar Pandey, Nikita Jadhav, Saiprasad Ajgaonkar, Dilip Mehta, Sujit Nair

**Affiliations:** Synergia Life Sciences Pvt. Ltd., Mumbai, India

**Keywords:** microRNA, neuropathic pain, biomarker, neuropathy, network, target, noncoding RNA

## Abstract

Neuropathic pain is a chronic pain condition seen in patients with diabetic neuropathy, cancer chemotherapy-induced neuropathy, idiopathic neuropathy as well as other diseases affecting the nervous system. Only a small percentage of people with neuropathic pain benefit from current medications. The complexity of the disease, poor identification/lack of diagnostic and prognostic markers limit current strategies for the management of neuropathic pain. Multiple genes and pathways involved in human diseases can be regulated by microRNA (miRNA) which are small non-coding RNA. Several miRNAs are found to be dysregulated in neuropathic pain. These miRNAs regulate expression of various genes associated with neuroinflammation and pain, thus, regulating neuropathic pain. Some of these key players include adenylate cyclase (*Ac9),* toll-like receptor 8 *(Tlr8),* suppressor of cytokine signaling 3 *(Socs3),* signal transducer and activator of transcription 3 *(Stat3)* and RAS p21 protein activator 1 *(Rasa1)*. With advancements in high-throughput technology and better computational power available for research in present-day pharmacology, biomarker discovery has entered a very exciting phase. We dissect the architecture of miRNA biological networks encompassing both human and rodent microRNAs involved in the development of neuropathic pain. We delineate various microRNAs, and their targets, that may likely serve as potential biomarkers for diagnosis, prognosis, and therapeutic intervention in neuropathic pain. miRNAs mediate their effects in neuropathic pain by signal transduction through IRAK/TRAF6, TLR4/NF-κB, TXIP/NLRP3 inflammasome, MAP Kinase, TGFβ and TLR5 signaling pathways. Taken together, the elucidation of the landscape of signature miRNA regulatory networks in neuropathic pain will facilitate the discovery of novel miRNA/target biomarkers for more effective management of neuropathic pain.

## Introduction

Neuropathic pain is defined as “pain induced by a lesion or disease of the somatosensory nervous system” by International Association for the Study of Pain (Bouhassira and Attal, 2019). The overall prevalence of neuropathy-derived pain in the general populace is 7–10%. Neuropathic pain is more frequently diagnosed in women (8%) as compared to men (5.7%) (Murphy et al., 2020). The prevalence of neuropathic pain in the United Kingdom and in the United States is 1 and 2% respectively (Smith and Torrance, 2012). Neuropathic pain affects 20–26.4% of diabetic patients (Bouhassira et al., 2013) and 20% of patients with herpes zoster in the United States (Johnson and Rice, 2014; Saguil et al., 2017). 48–74% patients with low back-related leg pain (Harrisson et al., 2020) and 40% of people after surgery suffer from neuropathic pain (Johansen et al., 2012). Studies have found that about 8.1–17.9% of the Canadian population is affected by neuropathic pain (VanDenKerkhof et al., 2016). Another study showed that the East Asian population has a low incidence of neuropathic pain (3.2%) (Inoue et al., 2017).

Peripheral neuropathic pain and central neuropathic pain are two different types of neuropathic pain. Postherpetic neuralgia, diabetic neuropathy, and causalgia are examples of peripheral neuropathic pain, caused by an injury or dysfunction in the PNS (peripheral nervous system) (McCarberg et al., 2017). Central neuropathic pain, such as thalamic pain, post-stroke pain, and post-spinal cord injury pain, is caused by an injury or dysfunction in the CNS (central nervous system) (Colloca et al., 2017). Neuropathic pain can also be divided into two types: stimulus-evoked and stimulus-independent. Mechanical, thermal, or chemical stimulation causes hyperalgesia and allodynia, which are indications of stimulus-evoked pain and stimulus-independent pain is typically categorized as shooting, stabbing, or burning (Kerstman et al., 2013).

Peripheral tissue injury in peripheral neuropathic pain results in release of inflammatory cytokines/mediators/chemokines [e.g., IL-1β, TGF-β, and chemokine (C-C motif) ligand 2 (CCL2)] as well as neurotrophic factors like nerve growth factor that sensitize nociceptors (Sun et al., 2021). This leads to dysregulated expression of ion channels in sensory neurons causing reduction of thermal and mechanical threshold of nociceptors which is known as peripheral sensitization. The abnormal excitation of peripheral neurons results in increased levels of neurotransmitters like substance P and glutamate in the spinal cord dorsal horn. This results in activation of neurokinin receptor and α-amino-3-hydroxy-5-methyl-4-isoxazolepropionic acid (AMPA)/N-Methyl-D-aspartic acid (NMDA) receptors causing long-lasting elevated excitability of dorsal horn neurons which is known as central sensitization (Woolf, 2011). Persistent stimulation of proinflammatory proteins and constant peripheral sensitization might induce central sensitization of spinal cord dorsal horn in diabetic neuropathic pain (Zhu D. et al., 2019).

Nociceptors are a type of sensory neurons which are triggered by noxious stimuli like heat, cold, mechanical force, or chemical stimulation. Based on the molecular mechanisms of nociception, nociceptors are classified as – thermal nociceptors, mechanical nociceptors, and chemical nociceptors (Tracey, 2017). Thermal nociceptors belong to transient receptor potential cation channel (TRP) receptor family of which the vanilloid variant (TRPV) is found in thermal nociceptive receptors that is responsible for thermal stimuli transduction. The TRPV family consists of TRPV1, TRPV2, TRPV3 and TRPV4 which are stimulated at various thermal temperatures or thermal stimuli (Frias and Merighi, 2016). Mechanical receptors or mechanoreceptors are stimulated by noxious mechanical force. While not much is known about mechanoreceptors, some inhibitory mechanoreceptors like (TWIK-related potassium) TREK and potassium voltage-gated channel subfamily A member 1 (Kv1.1) are known to be involved in nocifensive (defense against injury) behavior by maintaining the noxious mechanical force threshold significantly high to prevent hyperactivity of the mechanoreceptors (Armstrong and Herr, 2021). Chemical nociceptors are expressed by nociceptive neurons in response to harmful, noxious, or irritating chemicals. Apart from thermal stimuli, TRP receptor family can also detect noxious chemicals. While TRPV1 binds to prostaglandins, capsaicin, bradykinin, and other important pro-inflammatory molecules; TRPA1 subfamily is known to detect a wide variety of pungent and oily isothiocyanate compounds like cinnamaldehyde, mustard oils, and formaldehyde (Armstrong and Herr, 2021). Another example of noxious chemical receptors are the acid-sensing ion channels. They are stimulated by protons resulting in opening of their cation channels. They are found throughout the central as well as peripheral nervous system (St. John Smith, 2018).

In some individuals, there may be a possibility of a genetic basis for developing neuropathic pain. A study (Armero et al., 2012) conducted on the Caucasian population concluded that females with polymorphism in the TRPV1 gene are more susceptible to develop neuropathic pain. Met315Ile TRPV1 genotype only in females diagnosed with neuropathic pain, together with other physiological factors such as sex, might influence susceptibility to neuropathic pain (Armero et al., 2012). Kalfon et al. (2019) studied the association of single nucleotide polymorphism in transient receptor potential cation channel subfamily V member 1 (TRPV1) and nerve growth factor (NGF) and localized provoked vulvodynia and observed a significant relation between rs222747 of TRPV1 (c.945G>C, p.Ile315Met) and localized provoked vulvodynia in affected women. Genotyping analyses showed a critically high prevalence of polymorphism c.945G>C (rs222747) of TRPV1 and a single nucleotide polymorphism in the promoter region of NGF (rs11102930) in localized provoked vulvodynia women compared with controls. Substitution of the amino acid modifies the channel’s functional properties leading to increased TRPV1 protein expression because of an elevated copy number. The study further suggests that rs222747 “C” allele of TRPV1 to be a common genetic predisposition for other pain syndromes. Diabetic patients with a polymorphism in OPRM1 are susceptible to diabetic neuropathic pain (Zorina-Lichtenwalter et al., 2018). Black South Africans with polymorphism in KCNS1 are known to have HIV-associated sensory neuropathy (Zorina-Lichtenwalter et al., 2018).

One of the causes of neuropathic pain is the demyelination of peripheral nerve fibers. Demyelination destroys the molecular and structural features of the nerve fibers, which develops neuropathic pain (Wei et al., 2019). Vitamin K2 is believed to play a role in myelin repair and synthesis in the peripheral nervous system. Vitamin K2 activates and carboxylates Gla residues on GAS6 protein that is structurally related to anticoagulation factor protein S (vitamin K-dependent protein). GAS6, and protein S bind and activate the receptor tyrosine kinases of TAM (Tyro3, Axl, and Mer) family which increase myelin production as well as repair after myelin injury (Mehta, 2017). We have previously (Mehta et al., 2018) carried out an open-label observational study to determine the role of vitamin K2-7 in peripheral neuropathy associated with type-2 diabetic patients. In the 1st week, the visual analog score (VAS) score was 8–10 for T2DM patients. The VAS score had reduced to 1–2 in T2DM patients after treatment with vitamin K2-7 by the 12th week. The symptoms of PN had reduced persistently, which showed the effectiveness of vitamin K2-7 in management of peripheral neuropathy caused due to T2DM (Mehta et al., 2018). Proinflammatory cytokines such as TNFα and IL-1β are involved in neuropathic pain development through neuroinflammatory mechanisms (Li QY. et al., 2017). We have also demonstrated that vitamin K2-7 was able to inhibit TNFα and IL-1β gene expression in human monocyte-derived macrophages in a dose-dependent manner (Pan et al., 2016). In bones, primary activation of the RANKL/RANK (receptor activator for nuclear factor kappa B) system activates osteoclasts, which triggers the damage of bones and subsequently damages the peripheral sensory nerves around bones due to bone fracture. Damage to peripheral nerves can lead to neuropathic pain development (Zajączkowska et al., 2019). Activation of the RANKL/RANK system activates the nuclear factor kappa beta (NF-κB), an important regulator of inflammation, which leads to the activation of osteoclasts. Vitamin K2-7 prevents activation of the RANKL/RANK system by upregulating osteoprotegerin, a decoy receptor for RANKL. Vitamin K2-7 prevents the binding of RANKL to the RANK receptor which prevents activation of NF-κB and prevents activation of osteoclasts (Badmaev et al., 2011). Considering these properties of vitamin K2-7, it may likely serve as a potential therapeutic agent in the management of neuropathic pain.

MicroRNAs (miRNAs) are small single-stranded non-coding RNA molecules containing 19–25 nucleotides. miRNAs regulate almost every cellular process by regulating post-transcriptional gene expression and mRNA silencing (Bartel, 2018). miRNAs are found to be dysregulated in several diseases and regulate expression of various genes that are associated with different diseases. We have recently elucidated the non-coding RNA interactome including miRNA networks in cancer chemoprevention (Shah et al., 2021). We have also previously delineated the architecture of miRNA networks in mesothelioma (Gandhi and Nair, 2020), prostate adenocarcinoma (Nair et al., 2014; Nair and Kong, 2015a), cancer chemoprevention (Neelakandan et al., 2012; Nair and Kong, 2015b), as well as miRNA-lncRNA interactions (Nair, 2016). The term miRNA interactome includes cellular biomolecules, e.g., nucleic acids and proteins that interact with miRNA. In this review, we dissect the architecture of miRNA biological networks encompassing both human and rodent microRNAs involved in the development of neuropathic pain. We delineate various microRNAs, and their targets, that may likely serve as potential biomarkers for diagnosis, prognosis, and therapeutic intervention in neuropathic pain.

## Upregulated microRNAs in Neuropathic Pain

### Human microRNAs Upregulated in Neuropathic Pain

Leinders et al. (2016) investigated the role of hsa-miR-132-3p in white blood cells and sural nerve biopsies of patients with neuropathic pain. hsa-miR-132-3p was elevated in white blood cells as well as sural nerve biopsies of patients. In another study, Leinders et al. (2017) identified that hsa-miR-146a and hsa-miR-21 were upregulated in white blood cells of patients suffering from neuropathic pain. It was found that miR-21 was upregulated by 2.2-fold and hsa-miR-146a was upregulated by 10-fold. Li et al. (2017b) studied the expression of hsa-miR-199a-3p in patients with diabetic neuropathy (DN). hsa-miR-199a-3p was upregulated by ∼2.5-fold in patients with DN. Upregulation of hsa-miR-199a-3p inhibited extracellular serine protease inhibitor E2 (SerpinE2) expression. The downregulation of SerpinE2 by hsa-miR-199a-3p was thought to cause DN by boosting blood coagulation in the skin peripheral circulation. Tramullas et al. (2018) studied hsa-miR-30c-5p expression in individuals with neuropathic pain associated with leg ischemia. It was found that hsa-miR-30c-5p expression was increased in plasma and cerebrospinal fluid of neuropathic pain patients. Von Schack et al. (2011) used TaqMan Low Density Array (TLDA) and reported upregulation of hsa-miR-133b (10.2 fold). Heyn et al. (2016) conducted a study with patients suffering from neuropathic pain to determine the expression of miRNAs in neuropathic pain. Blood samples from neuropathic pain patients showed that hsa-miR-124a and hsa-miR-155 were upregulated by 2-fold. hsa-miR-124a and hsa-miR-155 reduced the expression of Sirtuin 1 (SIRT1) mRNA in patients, which led to the development of neuropathic pain. Thus, these miRNAs can be investigated as therapeutic targets in neuropathic pain.

Xu et al. (2014) conducted a study to determine the expressions of miRNAs in neuropathic pain. Using miRCURY LNA array, hsa-miR-22, hsa-miR-31-5p, and hsa-miR-133b were found to be upregulated by more than 2-fold. Hori et al. (2013) found that hsa-miR-28-3p and hsa-miR-223 were upregulated by more than 2 fold after miRNA expression profiling. Genda et al. (2013) studied the change in hsa-miR-124 expression neuropathic pain. hsa-miR-124 was upregulated by 2.26-fold. It was suggested that change in miRNA expression played a role in the maintenance and development of and therapy for neuropathic pain. Asahchop et al. (2018) conducted a study on HIV/AIDS patients diagnosed with symptomatic distal sensory polyneuropathy (sDSP). hsa-miR-455-3p was upregulated by 12-fold in HIV patients with sDSP as compared to non-sDSP HIV patients. Thus, hsa-miR-455-3p can be a potential biomarker for HIV-related sDSP. Chatterjee et al. (2018) conducted a study on individuals suffering from arsenic-induced peripheral neuropathy. It was found that hsa-miR-29a was upregulated by 3.63-fold in arsenic-exposed individuals with peripheral neuropathy. According to the finding, arsenic-induced peripheral neuropathy could be caused by the mir-29a/beta-catenin/PMP22 axis. Cao et al. (2019b) used skins of patients suffering from Postherpetic Neuralgia (PHN) to study the expression of miRNAs in PHN. hsa-miR-4491, hsa-miR-502-5p, hsa-miR-4528, hsa-miR-4721, hsa-miR-760, hsa-miR-495-3p, hsa-miR-382-5p, hsa-miR-4506, hsa-miR-1258, and hsa-miR-330-5p were upregulated by more than 5-fold in skin of PHN patients as compared to control. These miRNAs can be potential targets to treat PHN.

**FIGURE 1:**
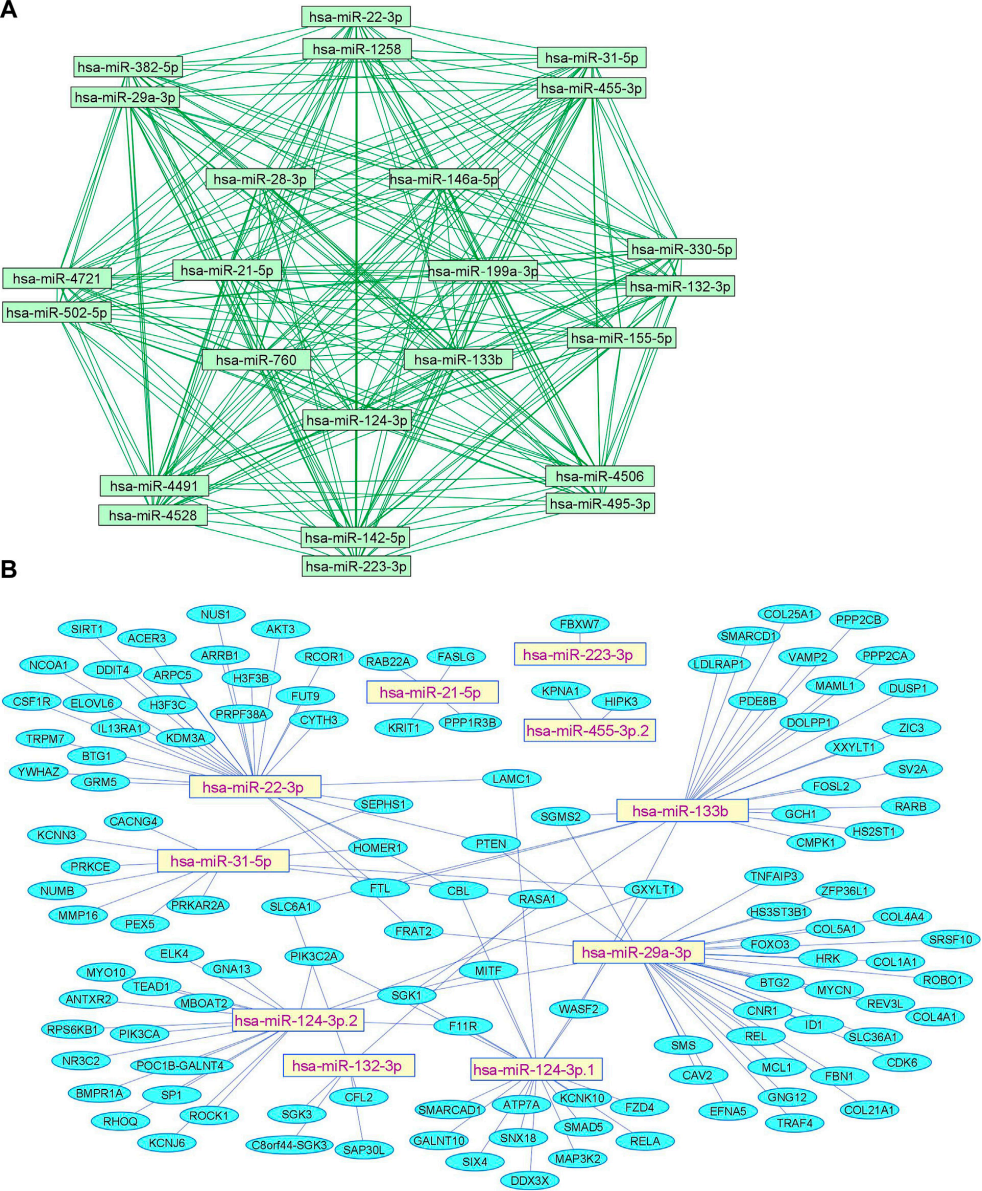
Upregulated human microRNA networks in neuropathic pain: **(A)** Human microRNA – microRNA network: Human miRNA-miRNA interaction network with 24 nodes and 276 edges. The networks were constructed using Cytoscape 3.8.2. **(B)** Human microRNA – gene target network: Architecture of networks of upregulated miRNAs in humans implicated in neuropathic pain showing interaction network of functionally enriched miRNAs with their targets with 137 nodes and 145 edges. The networks were constructed using Cytoscape 3.8.2. Using Mienturnet, the miRNA-target interactions were identified by TargetScan and the functionally enriched miRNAs were generated using the KEGG database.

We summarize upregulated human microRNAs in neuropathic pain in Table 1. An *in silico* method was used to construct miRNA-miRNA and miRNA-target networks of upregulated human miRNAs in neuropathic pain as shown in Figures 1A,B respectively*.*

**TABLE 1:** Upregulated human microRNAs involved in neuropathic pain.

Sr. No.	miRNA	Biological matrix (cell line/animal model/patient)	Targets	References
1	hsa-miR-132-3p	White blood cells from 81 patients with neuropathies of different etiologies	—	Leinders et al. (2016)
2	hsa-miR-155-5p hsa-miR-124-3p	CD4^+^ from neuropathic pain patients	SIRT1	Heyn et al. (2016)
3				
4	hsa-miR-199a-3p	Blood plasma from 60 patients with diabetic neuropathy	SERPINE2	Li et al. (2017b)
5	hsa-miR-455-3p	16 patients with symptomatic distal sensory polyneuropathy	NGF	Asahchop et al. (2018)
6	hsa-miR-29a-3p	Peripheral blood mononuclear cells from patients (*n* = 32) with arsenic-induced peripheral neuropathy	PMP22	Chatterjee et al. (2018)
7	hsa-miR-146a-5p	White blood cells from 76 patients with neuropathies of different etiologies	—	Leinders et al. (2017)
8	hsa-miR-21-5p			
9	hsa-miR-4491	Skin from 5 patients with postherpetic neuralgia	—	Cao et al. (2019b)
10	hsa-miR-502-5p			
11	hsa-miR-4528			
12	hsa-miR-4721			
13	hsa-miR-760			
14	hsa-miR-495-3p			
15	hsa-miR-382-5p			
16	hsa-miR-4506			
17	hsa-miR-1258			
18	hsa-miR-330-5p			
19	hsa-miR-142-5p	Human neuronal cell line (SH-SY5Y)	Soluble guanylate cyclase (sGC)	Xu et al. (2019a)
20	hsa-miR-28-3p	Chronic constriction injury (Sprague-Dawley rats) of sciatic nerve. Using TLDA MicroRNA cards v.3 A and B (contains human and rodent miRNAs)	—	Hori et al. (2013)
21	hsa-miR-223-3p			
22	hsa-miR-31-5p	Serum of Sprague-Dawley rats with spinal nerve ligation-induced neuropathic pain with microarray analysis performed using miRCURY LNA array ready to spot v.7.1 (contains human, mouse and rat miRNAs)	—	Xu et al. (2014)
23	hsa-miR-133b			
24	hsa-miR-22			

### Mouse microRNAs Upregulated in Neuropathic Pain

Gong et al. (2015) found that mmu-miR-98-5p and mmu-miR-210-3p were overexpressed in the diabetic neuropathic pain model of mice. Microarray studies showed that mmu-miR-98-5p regulated the Interleukin-6 (*Il-6*) gene. Thus, changes in miRNA level may affect inflammatory network homeostasis leading to the development of diabetic neuropathic pain. Jia et al. (2018) found out that overexpression of exosomal miRNA-28, -31a, and -130a in high glucose-stimulated Schwann cells of hyperglycemic mice led to the development of diabetic peripheral neuropathy (DPN). An increase in levels of exosomal miRNAs decreased the levels of proteins *Dnmt3a, Numb, Snap25,* and *Gap43*, which in turn led to the development of DPN. Exosomal miRNAs have been investigated as gene therapy for DPN. Thus, exosomes derived from mesenchymal stromal cells loaded with miR-146a (exo-146a) suppressed endothelial cell activation as well as peripheral blood inflammatory monocytes through inhibition of toll-like receptor (TLR)-4/NF-kappaB signaling pathway (Fan et al., 2021). Further, Numb is an endocytic protein that complexes with non-SUMOylated collapsin response mediator protein 2 (CRMP2) as well as E3 ubiquitin ligase Nedd4-2 and epidermal growth factor receptor pathway substrate 15 (Eps15). The complex then promotes clathrin-mediated endocytosis of voltage-gated sodium channels (Na_V_1.7) that plays a key role in neuronal excitability and neuropathic pain. It was observed that prevention of CRMP2 SUMOylation signaling pathway in CRMP2^K374A/K374A^ female mice having neuropathic pain reversed mechanical allodynia (Gomez et al., 2021) likely through Numb/CRMP2/Nedd4-2/Eps15 axis. Hu et al. (2019) observed that mmu-miR-34c was significantly upregulated in trigeminal ganglion tissue of type 1 diabetic mice. Upregulation of mmu-miR-34c decreased the expression of proteins Microtubule-associated protein 1A/1B-light chain 3-II (LC3-II) and Autophagy Related 4B Cysteine Peptidase (Atg4B), which affected corneal nerve regeneration in diabetes leading to diabetic corneal neuropathy. Peng et al. (2019b) studied that mmu-miR-124-3p was overexpressed in the chemotherapy-induced peripheral neuropathy (CiPN) mice model. The increase in mmu-miR-124-3p corresponded with cold allodynia and degeneration of axon in dorsal root ganglion and sciatic nerve, which contributed to the development of CiPN. Zhang et al. (2018C) showed that overexpression of mmu-miR-21 caused neuropathic pain in mice. Overexpression of mmu-miR-21 led to overexpression of Toll Like Receptor 8 (*Tlr8*). An increase in TLR8 expression mediated ERK activation, production of inflammatory mediators, and neuronal hyperexcitability leading to neuropathic pain. Cheng et al. (2015) identified that mmu-miR-341 was upregulated in mice with diabetic polyneuropathy. Antagonizing the elevated levels of mmu-miR-341 improved electrophysiological, structural, and behavioral abnormalities of sensory neurons caused due to neuropathy. These findings showed that mmu-miR-341 caused abnormalities in sensory neurons of mice which led to diabetic polyneuropathy. Hori et al. (2013) reported that mmu-miR-125a-5p, -132, -191, -222, -212 and -133a were upregulated by more than 2 fold. Wilkerson et al. (2020) found that mmu-miR-142-5p was upregulated (∼20 fold) in sciatic nerves of CCI mice. Hori et al. (2016) determined expression profiles of miRNAs in neuropathic pain. mmu-miR-449a, -18a, -130b, and -223 were upregulated by more than 2-fold in sciatic nerves of mice.

**FIGURE 2:**
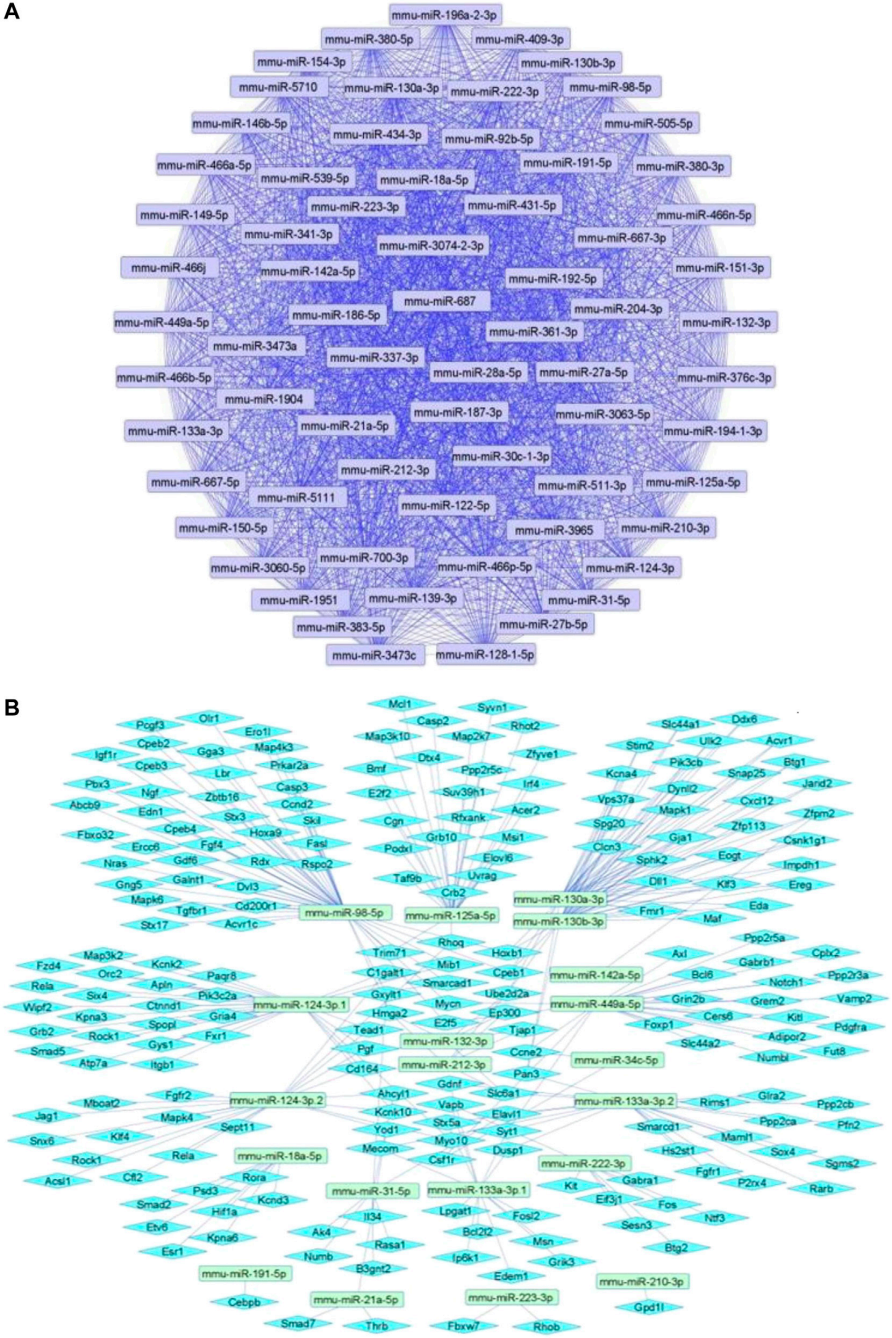
Upregulated mouse microRNA networks in neuropathic pain: **(A)** Mouse microRNA – microRNA network: Mouse miRNA-miRNA interaction network with 68 nodes and 2,278 edges. The networks were constructed using Cytoscape 3.8.2. **(B)** Mouse microRNA – gene target network: Architecture of networks of upregulated miRNAs in mouse implicated in neuropathic pain showing interaction network of functionally enriched miRNAs with their targets with 237 nodes and 287 edges. The networks were constructed using Cytoscape 3.8.2. Using Mienturnet, the miRNA-target interactions were identified by TargetScan and the functionally enriched miRNAs were generated using the KEGG database.

We summarize upregulated mouse microRNAs in neuropathic pain in Table 2. An *in silico* method was used to construct miRNA-miRNA and miRNA-target networks of upregulated mouse miRNAs in neuropathic pain as shown in Figures 2A,B respectively*.*

**TABLE 2:** Upregulated mouse microRNAs involved in neuropathic pain.

Sr. No.	miRNA	Biological matrix (cell line/animal model/patient)	Targets	References
1	mmu-miR-687	Spared-nerve injury of dorsal root ganglion of Kunming mice	—	Lu et al. (2017)
2	mmu-miR-139-3p			
3	mmu-miR-337-3p			
4	mmu-miR-124-3p	Dorsal root ganglion and sciatic nerve of paclitaxel-induced peripheral neuropathic C57BL/6 mice	—	Peng et al. (2019b)
5	mmu-miR-3965	Lumbar spinal dorsal horn of Balb/c mice with streptozocin-induced diabetic neuropathic pain	*Il-1β, Tnf-α, Il-13, Il-6,* and *Il-10*	Gong et al. (2015)
6	mmu-miR-3063-5p			
7	mmu-miR-466n-5p			
8	mmu-miR-505-5p			
9	mmu-miR-196a-2-3p			
10	mmu-miR-5710			
11	mmu-miR-466a-5p			
12	mmu-miR-466b-5p			
13	mmu-miR-3473a			
14	mmu-miR-3060-5p			
15	mmu-miR-466p-5p			
16	mmu-miR-187-3p			
17	mmu-miR-128-1-5p			
18	mmu-miR-3074-2-3p			
19	mmu-miR-210-3p			
20	mmu-miR-194-1-3p			
21	mmu-miR-27a-5p			
22	mmu-miR-667-3p			
23	mmu-miR-98-5p			
24	mmu-miR-28a-5p	Exosomes derived from schwann cells of C57L/J mice	*Dnmt3a, Gap43, Numb,* and *Snap25*	Jia et al. (2018)
25	mmu-miR-130a-3p			
26	mmu-miR-149-5p	Dorsal root ganglion of streptozocin-induced diabetic CD1 mice	—	Cheng et al. (2015)
27	mmu-miR-341-3p			
28	mmu-miR-21a-5p	Dorsal root ganglion of C57BL/6 and ICR mice with spinal nerve ligation-induced neuropathic pain	*Tlr8*	Zhang et al. (2018c)
29	mmu-miR-125a-5p	Chronic constriction injury (Sprague-Dawley rats) of sciatic nerve with microarray analysis performed using TLDA MicroRNA cards v.3 A and B (contains human and rodent miRNAs)	—	Hori et al. (2013)
30	mmu-miR-132-3p			
31	mmu-miR-151-3p			
32	mmu-miR-191-5p			
33	mmu-miR-222-3p			
34	mmu-miR-31-5p			
35	mmu-miR-434-3p			
36	mmu-miR-539-5p			
37	mmu-miR-133a-3p			
38	mmu-miR-150-5p			
39	mmu-miR-212-3p			
40	mmu-miR-383-5p			
41	mmu-miR-186-5p			
42	mmu-miR-122-5p	Dorsal root ganglion of C57BL/6 mice	—	Friedman et al. (2019)
43	mmu-miR-142a-5p	Spinal cord and sciatic nerve of C57BL/6 mice with chronic constriction injury-induced neuropathic pain	—	Wilkerson et al. (2020)
44	mmu-miR-431-5p	Dorsal root ganglion of C57BL/6 mice with partial sciatic nerve ligation-induced neuropathic pain	—	Hori et al. (2016)
45	mmu-miR-511-3p			
46	mmu-miR-204-3p			
47	mmu-miR-92b-5p			
48	mmu-miR-409-3p			
49	mmu-miR-154-3p			
50	mmu-miR-146b-5p			
51	mmu-miR-449a-5p			
52	mmu-miR-667-5p			
53	mmu-miR-434-3p			
54	mmu-miR-5111			
55	mmu-miR-700-3p			
56	mmu-miR-3473c			
57	mmu-miR-361-3p			
58	mmu-miR-27b-5p			
59	mmu-miR-18a-5p			
60	mmu-miR-30c-1-3p			
61	mmu-miR-376c-3p			
62	mmu-miR-192-5p			
63	mmu-miR-380-3p			
64	mmu-miR-223-3p			
65	mmu-miR-466j			
66	mmu-miR-130b-3p			
67	mmu-miR-1904	Rat lingual nerve tissue of Sprague-Dawley rats. Using TLDA Rodent miRNA Cards v.3 A and B	—	Tavares-Ferreira et al. (2019)
68	mmu-miR-1951			

### Rat microRNAs Upregulated in Neuropathic Pain

Li et al. (2021a) found that overexpression of rno-miR-142-3p developed neuropathic pain in chronic constriction injury (CCI) rat model. Adenylate cyclase 9 (*Ac9*) and cAMP levels were significantly reduced in CCI rats. A decrease in *Ac9* level led to an increase in expression of inflammatory factors *via* reduced expression of cAMP/AMPK pathway-related proteins, thereby, leading to the development of neuropathic pain. Sakai and Suzuki (2013) demonstrated that upregulation of rno-miR-21 contributed to neuropathic pain in rats. An increase in rno-miR-21 in dorsal root ganglion was responsible for pain development. In addition, intrathecal *Il-1β* elevated rno-miR-21 expression in the dorsal root ganglion, resulting in neuropathic pain. Sun et al. (2020) suggested that rno-miR-9 was overexpressed in sciatic nerves of rats with diabetes. Increased expression of ISL LIM homeobox 1 (*Isl1*) led to activation of sonic hedgehog (SHH) signaling pathway. rno-miR-9 contributed to diabetic peripheral neuropathy through the SHH signaling pathway by binding to ISL1. Wang et al. (2019) observed that rno-miR-195 expression was decreased in the infraorbital nerve CCI rat model. On the contrary, expression of Patched1 decreased notably. mmu-miR-195 aggravated neuropathic pain by activating the SHH signaling pathway by binding Patched1. Wei et al. (2020) found that rno-miR-24-3p expression was increased in CCI rats. rno-miR-24-3p activated Wnt5a/β-Catenin signaling by decreasing the expression of *Lpar3* to promote neuropathic pain development.

Li et al. (2019b) showed that rno-miR-15a and rno-miR-16 expression was remarkably increased in the CCI rat spinal cord. Thermal hyperalgesia and mechanical allodynia in CCI rats were reduced significantly when rno-miR-15a and rno-miR-16 were downregulated. G Protein-Coupled Receptor Kinase 2 (*Grk2*) was found to be a potential target of rno-miR-15a and rno-miR-16. Inhibition of rno-miR-15a and rno-miR-16 remarkably increased the expression of *Grk2* in CCI rats. Notably, the silencing of *Grk2* significantly reversed the effects of rno-miR-15a/16 downregulation. In conclusion, increased expression of rno-miR-15a and rno-miR-16 downregulated the expression of *Grk2* leading to the development of neuropathic pain. Yan et al. (2018a) studied that rno-miR-32-5p was upregulated in spinal microglia of rats after spinal nerve ligation. Dual Specificity Phosphatase 5 (*Dusp5*) was found to be a target of rno-miR-32-5p. rno-miR-32-5p promoted neuropathic pain development by downregulating the expression of *Dusp5*. Li and Zhao (2016) studied that expression of rno-miR-218 was upregulated in rats after CCI. Suppressor Of Cytokine Signaling 3 (*Socs3*), a critical inflammatory mediator, was a direct target of rno-miR-218. rno-miR-218 downregulated the expression of *Socs3*, thus, activating JAK/STAT3 inflammatory signaling which led to the development of neuropathic pain.

**FIGURE 3:**
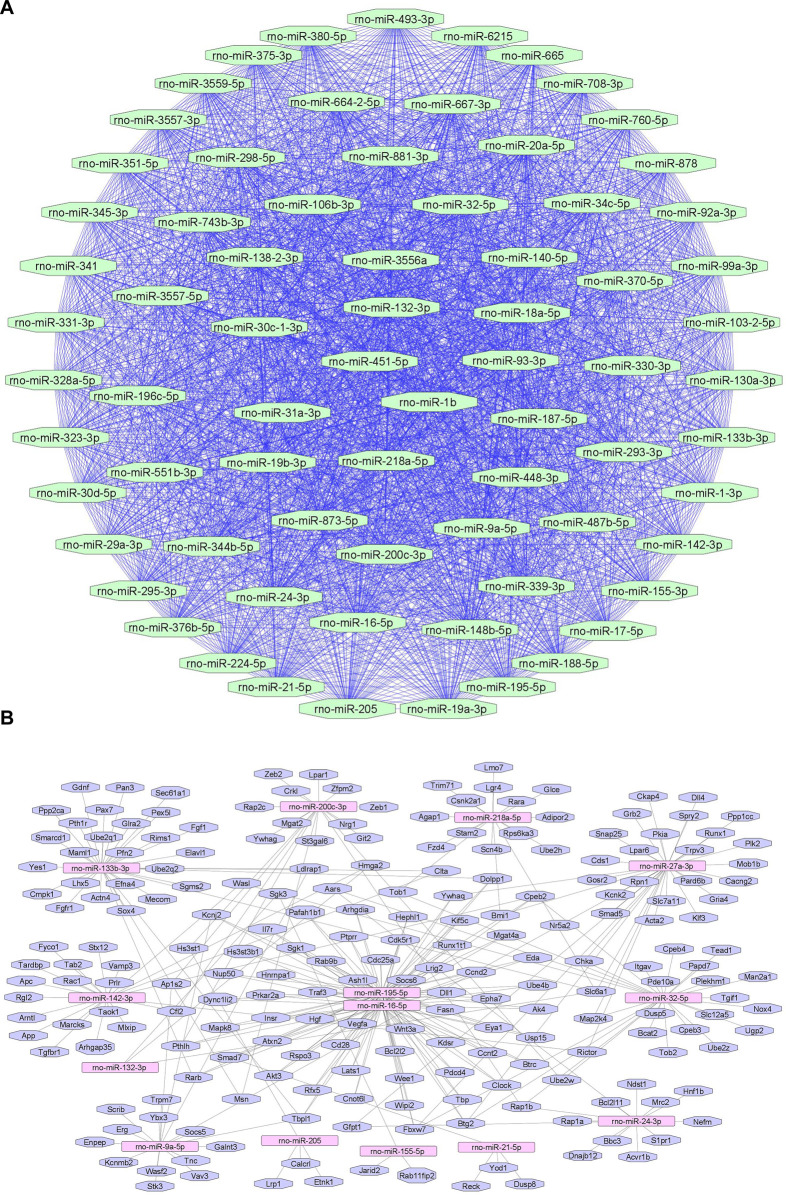
Upregulated rat microRNA networks in neuropathic pain: **(A)** Rat microRNA – microRNA network: Rat miRNA-miRNA interaction network with 73 nodes and 2,628 edges. The networks were constructed using Cytoscape 3.8.2. **(B)** Rat microRNA – gene target network: Architecture of networks of upregulated miRNAs in rat implicated in neuropathic pain showing interaction network of functionally enriched miRNAs with their targets with 205 nodes and 280 edges. The networks were constructed using Cytoscape 3.8.2. Using Mienturnet, the miRNA-target interactions were identified by TargetScan, and the functionally enriched miRNAs were generated using the KEGG database.

We summarize upregulated rat microRNAs in neuropathic pain in Table 3. An *in silico* method was used to construct miRNA-miRNA and miRNA-target networks of upregulated rat miRNAs in neuropathic pain as shown in Figures 3A,B respectively*.*

**TABLE 3:** Upregulated rat microRNAs involved in neuropathic pain.

Sr. No.	miRNA	Biological matrix (cell line/animal model/patient)	Targets	References
1	rno-miR-195-5p	Caudal brain stem of Wistar rats with infraorbital nerve chronic constriction injury-induced neuropathic pain	*Patched1*	Wang et al. (2019)
2	rno-miR-21-5p	Dorsal root ganglion of Sprague-Dawley rats with chronic constriction injury (CCI)-induced neuropathic pain	—	Sakai and Suzuki (2013)
3	rno-miR-140-5p	Dorsal root ganglion of Sprague-Dawley rats with CCI-induced neuropathic pain	—	Li et al. (2013)
4	rno-miR-341			
5	rno-miR-3559-5p			
6	rno-miR-760-5p			
7	rno-miR-200c-3p			
8	rno-miR-351-5p	Chronic constriction injury (Sprague-Dawley rats) of sciatic nerve	—	Hori et al. (2013)
9	rno-miR-345-3p			
10	rno-miR-339-3p			
11	rno-miR-19a-3p	Sciatic nerve of Sprague-Dawley rats with CCI-induced neuropathic pain	*Socs1*	Wang et al. (2015)
12	rno-miR-218a-5p	Spinal cord and microglial cells of Sprague-Dawley rats with CCI-induced neuropathic pain	*Socs3*	Li and Zhao (2016)
13	rno-miR-331-3p	Spinal dorsal horn of Sprague-Dawley rats with paclitaxel-induced neuropathic pain	—	Huang et al. (2016)
14	rno-miR-188-5p			
15	rno-miR-132-3p	Dorsal root ganglion and spinal cord of Holtzman rats with spared nerve injury-induced neuropathic pain	*Glua1, Glua2*	Leinders et al. (2016)
16	rno-miR-380-5p	Sciatic nerve of Sprague-Dawley rats with CCI-induced neuropathic pain	—	Ding et al. (2017)
17	rno-miR-205			
18	rno-miR-493-3p			
19	rno-miR-92a-3p	Dorsal root ganglion neurons of Sprague-Dawley rats with spinal nerve ligation-induced neuropathic pain	*Kcna1, Kcna4, Kcnc4, Kcnd3, Kcnq5, Dpp10, Scn1b*	Sakai et al. (2017)
20	rno-miR-17-5p			
21	rno-miR-19b-3p			
22	rno-miR-20a-5p			
23	rno-miR-18a-5p			
24	rno-miR-32-5p	Spinal microglial cells of Sprague-Dawley rats with spinal nerve ligation-induced neuropathic pain	*Dusp5*	Yan et al. (2018a)
25	rno-miR-30c-1-3p	Thalamus and anterior cingulate of Sprague-Dawley rats with complete brachial plexus avulsion-induced neuropathic pain	*Camk2b* and *Prkcg*	Liu et al. (2017b)
26	rno-miR-106b-3p			
27	rno-miR-93-3p			
28	rno-miR-873-5p			
29	rno-miR-451-5p	Dorsal root ganglion of Sprague-Dawley rats with streptozocin-induced diabetic neuropathy	—	Guo et al. (2018)
30	rno-miR-743b-3p			
31	rno-miR-881-3p			
32	rno-miR-330-3p	Dorsal spinal horn of Sprague-Dawley rats with CCI-induced neuropathic pain	—	Peng et al. (2019a)
33	rno-miR-16-5p	Lumbar spinal cord of Sprague-Dawley rats with CCI-induced neuropathic pain	*Grk2*	Li et al. (2019b)
34	rno-miR-667-3p	Rat lingual nerve tissue of Sprague-Dawley rats	—	Tavares-Ferreira et al. (2019)
35	rno-miR-1-3p	Dorsal spinal cord of Sprague-Dawley rats with CCI-induced neuropathic pain	—	Cao et al. (2019a)
36	rno-miR-376b-5p			
37	rno-miR-31a-3p			
38	rno-miR-1b			
39	rno-miR-448-3p	Spinal cord of Sprague-Dawley rats with CCI-induced	*Sirt1*	Chu et al. (2019)
40	rno-miR-34c-5p	Sciatic nerve of Sprague-Dawley rats with CCI-induced neuropathic pain	*Sirt1*	Mo et al. (2020)
41	rno-miR-155-3p	Schwann cells of Sprague-Dawley rats with streptozocin-induced diabetic peripheral neuropathy	—	Wang et al. (2020a)
42	rno-miR-224-5p			
43	rno-miR-99a-3p			
44	rno-miR-142-3p	Sciatic nerve of Sprague-Dawley rats with CCI-induced neuropathic pain	*Ac9*	Li et al. (2021a)
45	rno-miR-30d-5p	Sciatic nerve of Sprague-Dawley rats with streptozocin-induced diabetic neuropathy	*Isl1*	Sun et al. (2020)
46	rno-miR-29a-3p			
47	rno-miR-375-3p			
48	rno-miR-9a-5p			
49	rno-miR-133b-3p	Sciatic nerve tissue of Sprague-Dawley rats with streptozocin-induced diabetic neuropathy	—	Chang et al. (2020)
50	rno-miR-103-2-5p	Sciatic nerve of Sprague-Dawley rats with streptozocin-induced diabetic neuropathy	—	Li et al. (2020c)
51	rno-miR-138-2-3p			
52	rno-miR-148b-5p			
53	rno-miR-187-5p			
54	rno-miR-196c-5p			
55	rno-miR-293-3p			
56	rno-miR-295-3p			
57	rno-miR-298-5p			
58	rno-miR-323-3p			
59	rno-miR-328a-5p			
60	rno-miR-344b-5p			
61	rno-miR-3556a			
62	rno-miR-3557-3p			
63	rno-miR-3557-5p			
64	rno-miR-370-5p			
65	rno-miR-487b-5p			
66	rno-miR-551b-3p			
67	rno-miR-6215			
68	rno-miR-664-2-5p			
69	rno-miR-665			
70	rno-miR-708-3p			
71	rno-miR-878			
72	rno-miR-24-3p	Chronic constriction injury in Sprague-Dawley rats	*Lpar3*	Wei et al. (2020)
73	rno-miR-130a-3p	Spinal cord of rats with spinal cord injury-induced neuropathic pain	*Igf-1*	Yao et al. (2021)

## Downregulated microRNAs in Neuropathic Pain

### Human microRNAs Downregulated in Neuropathic Pain

Liu et al. (2019) found that hsa-miR-101 expression was found to be decreased in plasma and sural nerve biopsies from patients with neuropathic pain. Reduction in hsa-miR-101 led to Nuclear Factor Kappa B (NF-κB) activation which contributed to the development of neuropathic pain. Von Schack et al. (2011) studied the expressions of miRNAs using TLDA miRNA panel. hsa-miR-103, hsa-miR-181b, hsa-miR-137, hsa-miR-23b, hsa-miR-26b, hsa-miR-148a, hsa-miR-181c, hsa-miR-148b, hsa-miR-125b, hsa-miR-133a, hsa-let-7a, hsa-let-7b, hsa-let-7c, hsa-let-7d, hsa-let-7e, hsa-let-7g, hsa-miR-10a, hsa-miR-497, hsa-miR-93, hsa-miR-10b, hsa-miR-21 and hsa-miR-34a were downregulated more than 2 fold. Cao et al. (2019b) collected skin samples of patients suffering from Postherpetic Neuralgia (PHN) to study the miRNA expression profile. More than 10-fold downregulation of following miRNAs was observed: hsa-miR-4772-5p, hsa-miR-2682-5p, hsa-miR-3678-3p, hsa-miR-3678-5p, hsa-miR-5579-3p, hsa-miR-3664-3p, hsa-miR-4692, hsa-miR-4680-3p, hsa-miR-3187-3p, and hsa-miR-518e-3p. These miRNAs could be potential targets for treating PHN.

**FIGURE 4:**
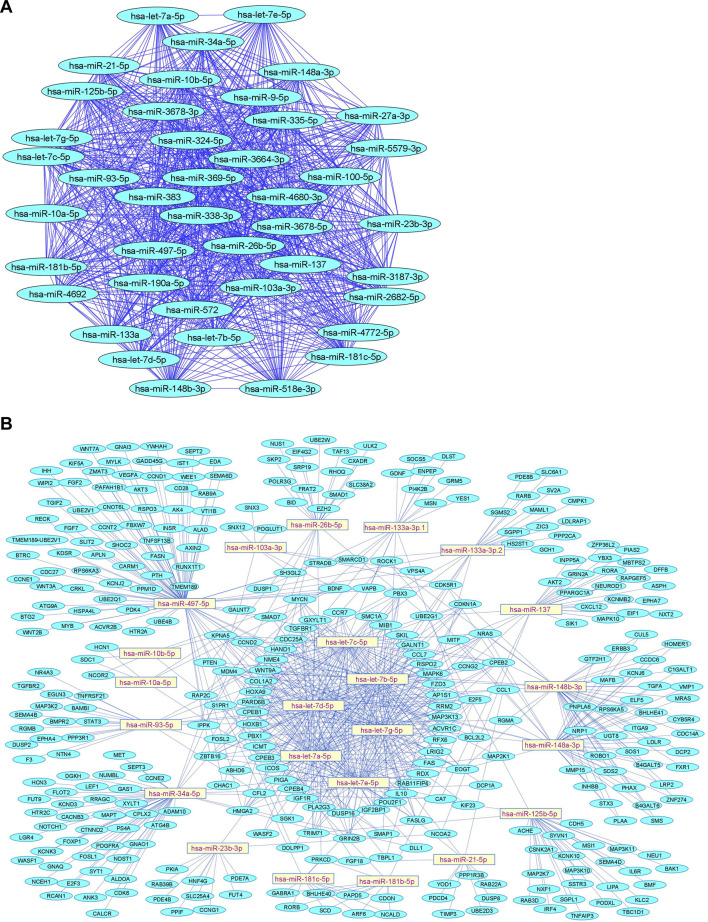
Downregulated human microRNA networks in neuropathic pain: **(A)** Human microRNA – microRNA network: Human miRNA-miRNA interaction network with 42 nodes and 861 edges. The networks were constructed using Cytoscape 3.8.2. **(B)** Human microRNA – gene target network: Architecture of networks of upregulated miRNAs in humans implicated in neuropathic pain showing interaction network of functionally enriched miRNAs with their targets with 380 nodes and 759 edges. The networks were constructed using Cytoscape 3.8.2. Using Mienturnet, the miRNA-target interactions were identified by TargetScan and the functionally enriched miRNAs were generated using the KEGG database.

We summarize downregulated human microRNAs in neuropathic pain in Table 4. An *in silico* method was used to construct miRNA-miRNA and miRNA-target networks of downregulated human miRNAs in neuropathic pain as shown in Figures 4A,B respectively*.*

**TABLE 4:** Downregulated human microRNAs involved in neuropathic pain.

Sr. No.	miRNA	Biological matrix (cell line/animal model/patient)	Targets	References
1	hsa-miR-4772-5p	Skin from 5 patients with postherpetic neuralgia	—	Cao et al. (2019b)
2	hsa-miR-2682-5p			
3	hsa-miR-3678-3p			
4	hsa-miR-3678-5p			
5	hsa-miR-5579-3p			
6	hsa-miR-3664-3p			
7	hsa-miR-4692			
8	hsa-miR-4680-3p			
9	hsa-miR-3187-3p			
10	hsa-miR-518e-3p			
11	hsa-miR-34a-5p	Dorsal root ganglia of Sprague-Dawley rats with spinal nerve ligation induced neuropathic pain using TLDA Human miRNA Panel	—	von Schack et al. (2011)
12	hsa-let-7e-5p			
13	hsa-let-7a-5p			
14	hsa-miR-21-5p			
15	hsa-miR-10b-5p			
16	hsa-let-7d-5p			
17	hsa-miR-93-5p			
18	hsa-miR-497-5p			
19	hsa-let-7b-5p			
20	hsa-miR-10a-5p			
21	hsa-let-7c-5p			
22	hsa-let-7g-5p			
23	hsa-miR-324-5p			
24	hsa-miR-133a			
25	hsa-miR-125b-5p			
26	hsa-miR-27a-3p			
27	hsa-miR-148b-3p			
28	hsa-miR-369-5p			
29	hsa-miR-181c-5p			
30	hsa-miR-100-5p			
31	hsa-miR-148a-3p			
32	hsa-miR-383			
33	hsa-miR-9-5p			
34	hsa-miR-26b-5p			
35	hsa-miR-190a-5p			
36	hsa-miR-23b-3p			
37	hsa-miR-137			
38	hsa-miR-181b-5p			
39	hsa-miR-335-5p			
40	hsa-miR-103a-3p			
41	hsa-miR-572			
42	hsa-miR-338-3p			

### Mouse microRNAs Downregulated in Neuropathic Pain

Zhu et al. (2019a) observed that mmu-miR-138 was decreased in mice after sciatic nerve injury. Decreased levels of mmu-miR-138 activated NF-κB pathway and inflammatory responses during nerve injury. Nerve injury resulted in neuropathic pain and subsequent pain hypersensitivity. Using the CCI model of neuropathic pain, Zhang et al. (2020b) found that mmu-miR-144 was downregulated after inducing CCI. Proinflammatory mediators like *Il-6, Il-1β* and *Tnfα* were significantly elevated after CCI induction. RAS P21 Protein Activator 1 (*Rasa1*), the target gene of mmu-miR-144, increased the levels of proinflammatory mediators after CCI. These results showed successful establishment of neuropathic pain in CCI-induced mice. Zhang et al. (2018a) observed that mmu-miR-25 was decreased in mice with diabetic peripheral neuropathy. Inhibition of mmu-miR-25 increased the levels of reactive oxygen species and overexpression of NADPH Oxidase 4 (*Nox4*). This imbalance was sufficient to aggravate the Schwann cells damage in sciatic nerves of diabetic mice, leading to diabetic peripheral neuropathy. Wang et al. (2020b) reported that a decrease in mmu-miR-27a in the exosomes derived from Schwann cells led to peripheral neuropathy in diabetic mice. A decrease in mmu-miR-27a led to the dysfunction of interaction between the axons and blood vessels that regulate peripheral nerve function, thus, contributing to diabetic peripheral neuropathy development. Wu et al. (2019) observed that expression of mmu-miR-193a was decreased in diabetic neuropathic pain. Downregulation of mmu-miR-193a increased the activity of inflammatory mediator protein High Mobility Group Box 1 (*Hmgb1*) which led to the progression of diabetic neuropathic pain.

Pan et al. (2018) observed that mmu-miR-23a was decreased in the pSNL model of neuropathic pain in mice. CXC chemokine receptor type 4 (*Cxcr4*) activity was increased and it targeted TXNIP/NLRP3 inflammasome axis to induce neuropathic pain in mice. Imai et al. (2011) observed that mmu-miR-200b and mmu-miR-429 expressions were decreased in nucleus accumbens neurons after sciatic nerve ligation in mice. Since Dnmt3a is a target of mmu-miR-200b and mmu-miR-429, the expression of DNA methyltransferase 3 alpha (Dnmt3a) was increased in nucleus accumbens. Double-immunolabeling with antibodies specific to Dnmt3a showed that increased Dnmt3a proteins were dominantly expressed in postsynaptic neurons in the nucleus accumbens area under a neuropathic pain-like state. Xu et al. (2019b) studied that mmu-miR-34c was downregulated in the CCI model of neuropathic pain. This led to an increase in inflammatory mediator *Nlrp3* expression, which led to the development of neuropathic pain. Wilkerson et al. (2020) conducted a study on CNS tissues of a mouse model of neuropathic pain to elucidate microRNAs expressed in neuropathic pain. mmu-miR-182-5p, mmu-miR-96-5p and mmu-miR-183-5p were downregulated in CCI mice with mechanical allodynia. It was found that this microRNA cluster regulates more than 80% of genes related to neuropathic pain. Wu et al. (2017) determined that expression of mmu-miR-106a was decreased in mice with diabetic peripheral neuropathy. Increased 12/15-LOX expression induced mechanical allodynia and thermal hyperalgesia in mice. 12/15-LOX was observed to be a target of mmu-miR-106a. Hence, downregulation of mmu-miR-106a led to the progression of neuropathic pain. Lu et al. (2017) studied miRNA expression profile using microarray analysis and found that mmu-miR-101a and mmu-miR-365-3p were downregulated in mice.

**FIGURE 5:**
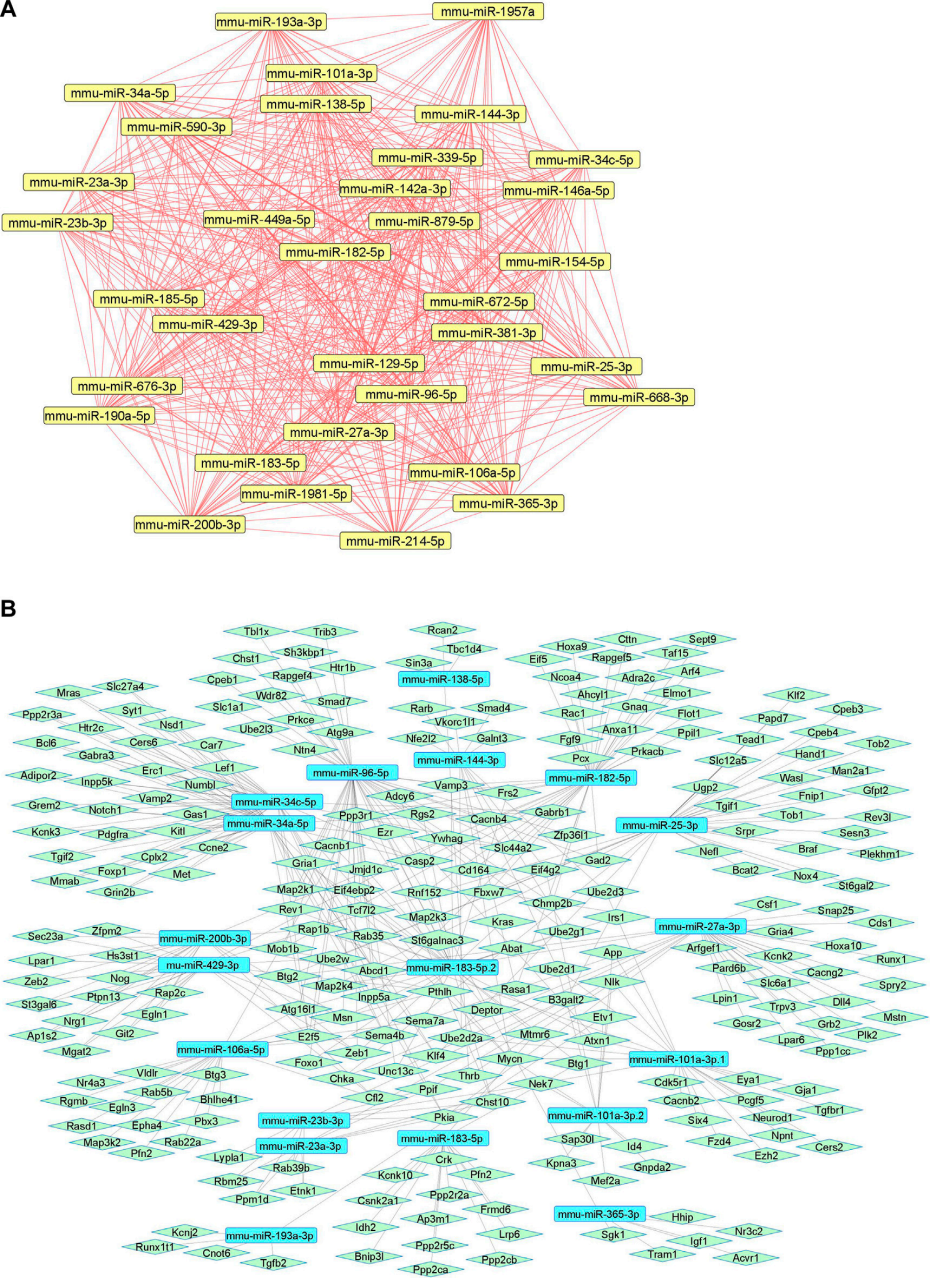
Downregulated mouse microRNA networks in neuropathic pain: **(A)** Mouse microRNA – microRNA network: Mouse miRNA-miRNA interaction network with 34 nodes and 561 edges. The networks were constructed using Cytoscape 3.8.2. **(B)** Mouse microRNA – gene target network: Architecture of networks of upregulated miRNAs in mouse implicated in neuropathic pain showing interaction network of functionally enriched miRNAs with their targets with 275 nodes and 413 edges. The networks were constructed using Cytoscape 3.8.2. Using Mienturnet, the miRNA-target interactions were identified by TargetScan and the functionally enriched miRNAs were generated using the KEGG database.

We summarize downregulated mouse microRNAs in neuropathic pain in Table 5. An *in silico* method was used to construct miRNA-miRNA and miRNA-target networks of downregulated mouse miRNAs in neuropathic pain as shown in Figures 5A,B respectively*.*

**TABLE 5:** Downregulated mouse microRNAs involved in neuropathic pain.

Sr. No.	miRNA	Biological matrix (cell line/animal model/patient)	Targets	References
1	mmu-miR-200b-3p	Nucleus accumbens of C57BL/6J mice with sciatic nerve ligation-induced neuropathic pain	Dnmt3a	Imai et al. (2011)
2	mmu-miR-429-3p			
3	mmu-miR-879-5p	Spinal dorsal horn of Sprague-Dawley rats with CCI-induced neuropathic pain with microarray analysis performed using Affymetrix 3.0 GeneChip miRNA Array (contains human and rodent miRNAs)	—	Genda et al. (2013)
4	mmu-miR-129-5p			
5	mmu-miR-23b-3p	Spinal cord injury-induced neuropathic pain in ICR mice	*Nox4*	Im et al. (2012)
6	mmu-miR-146a-5p	Sciatic nerve tissue of BKS.Cg-m+/+Leprdb/J (db/db) mice with diabetic peripheral neuropathy	*Traf6*	Liu et al. (2017a)
7	mmu-miR-1981-5p	Dorsal root ganglion of C57BL/6 mice with partial sciatic nerve ligation-induced neuropathic pain	—	Hori et al. (2016)
8	mmu-miR-214-5p			
9	mmu-miR-668-3p	Dorsal root ganglion of Sprague-Dawley rats with spinal nerve ligation-induced neuropathic pain with analysis performed using OneArray^®^ Mouse & Rat miRNA Microarray v4 chip	*Mapk1* and *Tead1*	Chang et al. (2017)
10	mmu-miR-672-5p			
11	mmu-miR-106a-5p	Dorsal root ganglion of mice with Streptozocin-induced diabetic peripheral neuropathy	*Alox15*	Wu et al. (2017)
12	mmu-miR-449a-5p	Spared-nerve injury of dorsal root ganglion of Kunming mice	*Trpa1*, *Kcnma1* and *Tpte*	Lu et al. (2017)
13	mmu-miR-365-3p			
14	mmu-miR-101a-3p			
15	mmu-miR-339-5p			
16	mmu-miR-185-5p			
17	mmu-miR-190a-5p	Lumbar spinal dorsal horn of Balb/c mice with Streptozocin-induced diabetic neuropathic pain	*Slc17a6*	Yang et al. (2017a)
18	mmu-miR-142a-3p	Dorsal root ganglion of ICR mice with spinal nerve ligation-induced neuropathic pain	*Hmgb1*	Zhang et al. (2017)
19	mmu-miR-23a-3p	Spinal glial cells of C57BL/6J mice with spinal nerve ligation-induced neuropathic pain	*Cxcr4*	Pan et al. (2018)
20	mmu-miR-34a-5p	Blood of complete Freund’s adjuvant-induced inflammatory pain model of C57BL/6 mice	*Xist*	Shenoda et al. (2018)
21	mmu-miR-25-3p	Schwann cells of Balb/c mice with diabetic neuropathy	*Nox4*	Zhang et al. (2018a)
22	mmu-miR-34c-5p	Spinal cord of C57BL/6 mice with chronic constriction injury-induced neuropathic pain	*Nlrp3*	Xu et al. (2019b)
23	mmu-miR-381-3p	Sciatic nerves of C22 mice with Charcot-Marie tooth disease type 1A	*Pmp22*	Lee et al. (2019)
24	mmu-miR-1957a	Rat lingual nerve tissue of Sprague-Dawley rats using TLDA Rodent miRNA Cards v.3 A and B	—	Tavares-Ferreira et al. (2019)
25	mmu-miR-193a-3p	Lumbar spinal dorsal horn of Balb/c mice with Streptozocin-induced diabetic neuropathic pain	*Hmgb1*	Wu et al. (2019)
26	mmu-miR-144-3p	Dorsal root ganglion of C57BL/6 mice with chronic constriction injury-induced neuropathic pain	*Rasa1*	Zhang et al. (2020b)
27	mmu-miR-138-5p	Spinal cord of C57BL/6 mice with sciatic nerve injury-induced neuropathic pain	NF-κB	Zhu et al. (2019a)
28	mmu-miR-27a-3p	Schwann cell exosomes of BKS.Cg-m1/1Leprdb/J (db/db) mice with diabetic peripheral neuropathy	—	Wang et al. (2020b)
29	mmu-miR-154-5p	Dorsal root ganglion of C57BL/6 mice with spinal nerve ligation-induced neuropathic pain	*Cxcl13*	Chen et al. (2020)
30	mmu-miR-676-3p	Spinal cord and sciatic nerve of C57BL/6 mice with chronic constriction injury-induced neuropathic pain	—	Wilkerson et al. (2020)
31	mmu-miR-182-5p			
32	mmu-miR-183-5p			
33	mmu-miR-96-5p			
34	mmu-miR-590-3p	Dorsal root ganglion tissue of db/db mice with diabetic peripheral neuropathy	*Rap1a*	Wu et al. (2020b)

### Rat microRNAs Downregulated in Neuropathic Pain

Zhong et al. (2019) found out that rno-miR-98 was downregulated in the CCI rat model. The study indicated that Signal Transducer and Activator of Transcription 3 (*Stat3*) was overexpressed, and it was found to be a probable target of rno-miR-98. Overexpression of *Stat3* led to an increase in Il-6, Il-1β, and Tnfα expression, which contributed to development of neuropathic pain. Zhang et al. (2019b) observed that rno-miR-124-3p was remarkably downregulated in rats after CCI. The expression of Il-6, Il-1β, and Tnfα proteins increased greatly after CCI, which contributed to neuroinflammation. Downregulation of rno-miR-124-3p led to an increase in expression of Enhancer of zeste homolog 2 (*Ezh2*), a direct target of rno-miR-124-3p. Overexpression of *Ezh2* and other inflammatory mediators led to the development of neuropathic pain. Li et al. (2020b) studied the effect of rno-miR-22-3p downregulation on the development of neuropathic pain. Downregulation of rno-miR-22-3p promoted the progression of neuropathic pain by regulating inflammation factors expression by directly targeting Enolase 1 (*Eno1*). Xie et al. (2020) found that rno-miR-101 was downregulated in lumbar spinal dorsal horns after CCI. mTOR (mRNA) was upregulated after CCI and was found to be a direct target of rno-miR-101. Activation of the mTOR signaling pathway was responsible for the onset, progression, and maintenance of neuropathic pain. Yan et al. (2017) observed that rno-miR-93 was downregulated in the spinal cord of CCI rats. *Stat3* expression was upregulated. It was found that *Stat3* was a direct target of rno-miR-93. Overexpression of rno-miR-93 remarkably reduced the expression of *Stat3 in vitro* and *in vivo*. Further, overexpression of *Stat3* markedly reversed the rno-miR-93 overexpression-induced repressive effects on neuropathic pain development and neuroinflammation. In conclusion, the downregulation of rno-miR-93 and upregulation of *Stat3* led to the development of neuropathic pain.

Zhang et al. (2018b) observed a significant decrease of rno-miR-26a-5p expression in the spinal cord tissues from CCI rats. Mitogen-Activated Protein Kinase 6 (*Mapk6*) was upregulated in CCI rats and was found to be a downstream target of rno-miR-26a-5p. *Mapk6* upregulation led to the progression of neuropathic pain. Zhang et al. (2020c) found that rno-miR-128-3p was significantly downregulated in the spinal cord of CCI rats. Zinc Finger E-Box Binding Homeobox 1 (*Zeb1*), an inflammation mediator, was upregulated in CCI rats and was found to be a target of rno-miR-128-3p. Upregulated *Zeb1* contributed to the development of neuropathic pain by promoting neuroinflammation. Miao et al. (2020) observed that rno-miR-183 was downregulated in the spinal dorsal horn of the CCI rat. *Hdac2* reduced the expression of rno-miR-183 by deacetylating histone H4. By upregulating *Hdac2* and activating the TXNIP-NLRP3 inflammasome axis, NF-κB p65 suppressed rno-miR-183 expression and generated an inflammatory response in rats, worsening neuropathic pain. Zhang et al. (2015) observed that rno-miR-141 expression was markedly decreased in CCI rats. Downregulation of rno-miR-141 led to an increase in the expression of *Hmgb1*. In CCI rats, *Hmgb1* overexpression exacerbated mechanical allodynia and thermal hyperalgesia, as well as increased proinflammatory cytokines. This led to the development of neuropathic pain in rats. Yan et al. (2018b) found out that rno-miR-200b and rno-miR-429 were notably downregulated in CCI rat spinal cords. *Zeb1* was predicted as the target of rno-miR-200b and rno-miR-429. *Zeb1* expression was significantly increased in CCI rats, and overexpression of rno-miR-200b and rno-miR-429 significantly inhibited *Zeb1* mRNA expression in rats. Knockdown of *Zeb1* reduced neuropathic pain development. The findings suggested that rno-miR-200b/rno-miR-429, through targeting *Zeb1*, could be an essential regulator of neuropathic pain development. Sakai et al. (2013) observed that rno-miR-7a expression decreased in rats with neuropathic pain. β2 subunit of the voltage-gated sodium channel was found to be a target of rno-miR-7a. β2 subunit protein expression was increased in the dorsal root ganglion of rats, which led to the development of neuropathic pain.

**FIGURE 6:**
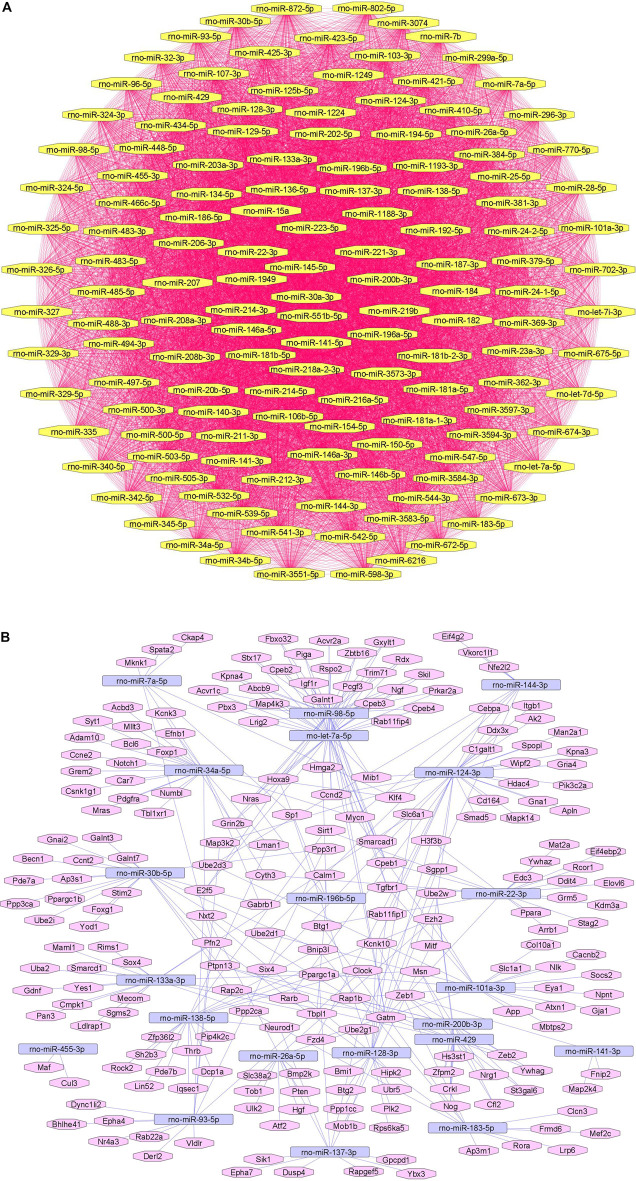
Downregulated rat microRNA networks in neuropathic pain: **(A)** Rat microRNA – microRNA network: Rat miRNA-miRNA interaction network with 140 nodes and 9,730 edges. The networks were constructed using Cytoscape 3.8.2. **(B)** Rat microRNA – gene target network: Architecture of networks of upregulated miRNAs in rats implicated in neuropathic pain showing interaction network of functionally enriched miRNAs with their targets with 239 nodes and 330 edges. The networks were constructed using Cytoscape 3.8.2. Using Mienturnet, the miRNA-target interactions were identified by TargetScan and the functionally enriched miRNAs were generated using the KEGG database.

We summarize downregulated rat microRNAs in neuropathic pain in Table 6. An *in silico* method was used to construct miRNA-miRNA and miRNA-target networks of downregulated rat miRNAs in neuropathic pain as shown in Figures 6A,B respectively*.*

**TABLE 6:** Downregulated rat microRNAs involved in neuropathic pain.

Sr. No.	miRNA	Biological matrix (cell line/animal model/patient)	Targets	References
1	rno-miR-183-5p	Dorsal root ganglion of Sprague-Dawley rats with spinal nerve ligation-induced neuropathic pain	Tia1	Aldrich et al. (2009)
2	rno-miR-96-5p			
3	rno-miR-103-3p	Spinal nerve of Wistar rats with spinal nerve ligation-induced neuropathic pain	*Cacna1c*, *Cacna2d1* and *Cacnb1*	Favereaux et al. (2011)
4	rno-miR-421-5p	Spinal dorsal horn of Sprague-Dawley rats with CCI-induced neuropathic pain	—	Genda et al. (2013)
5	rno-miR-207			
6	rno-miR-410-5p	Dorsal root ganglion of Sprague-Dawley rats with CCI-induced neuropathic pain	—	Li et al. (2013)
7	rno-miR-3583-5p			
8	rno-miR-146a-3p			
9	rno-miR-3597-3p			
10	rno-miR-598-3p			
11	rno-miR-541-3p			
12	rno-let-7a-5p			
13	rno-miR-196b-5p			
14	rno-miR-872-5p			
15	rno-miR-181a-1-3p			
16	rno-miR-218a-2-3p			
17	rno-miR-3584-3p			
18	rno-miR-434-5p			
19	rno-miR-485-5p			
20	rno-miR-466c-5p			
21	rno-miR-425-3p			
22	rno-miR-187-3p			
23	rno-miR-34b-5p			
24	rno-miR-28-5p			
25	rno-miR-221-3p			
26	rno-miR-448-5p			
27	rno-miR-324-3p			
28	rno-miR-7a-5p	Dorsal root ganglion of Sprague-Dawley rats with spinal nerve ligation-induced neuropathic pain	*Nefl*	Yang et al. (2019)
29	rno-miR-203a-3p	Spinal dorsal horn of Sprague-Dawley rats with bilateral CCI-induced neuropathic pain	Rap1a	Li et al. (2015)
30	rno-miR-141-3p	Dorsal root ganglion of Sprague-Dawley rats with CCI-induced neuropathic pain	*Hmgb1*	Zhang et al. (2015)
31	rno-miR-30b-5p	Dorsal root ganglion of Sprague-Dawley rats with nerve injury-induced neuropathic pain	*Scn9a*	Shao et al. (2016)
32	rno-miR-145-5p	Dorsal root ganglion of Sprague-Dawley rats with CCI-induced neuropathic pain	*Akt3*	Shi et al. (2018)
33	rno-miR-128-3p	Microglial cells of Sprague-Dawley rats with spinal cord injury-induced neuropathic pain	*Cd86*, *Cd32*, *Arg1*, and *Cd206*	Yang et al. (2017b)
34	rno-miR-206-3p	Microglial cells of Sprague-Dawley rats with CCI-induced neuropathic pain	*Zeb2*	Chen et al. (2019)
35	rno-miR-93-5p	Spinal cord of Sprague-Dawley rats with CCI-induced neuropathic pain	*Stat3*	Yan et al. (2017)
36	rno-miR-494-3p	Spinal cord of Sprague-Dawley rats with spinal cord injury-induced neuropathic pain	*Pten*	Gu et al. (2017)
37	rno-miR-192-5p	Sciatic nerve of Sprague-Dawley rats with CCI-induced neuropathic pain	—	Ding et al. (2017)
38	rno-miR-144-3p			
39	rno-miR-327			
40	rno-miR-296-3p			
41	rno-miR-539-5p			
42	rno-miR-505-3p			
43	rno-miR-214-3p			
44	rno-miR-184	Spared nerve injury-induced neuropathic pain in Sprague-Dawley rats	—	Zhou et al. (2017)
45	rno-miR-150-5p	Microglial cells of Sprague-Dawley rats with CCI-induced neuropathic pain	*Tlr5*	Ji et al. (2018)
46	rno-miR-137-3p	Microglial cells of Sprague-Dawley rats with CCI-induced neuropathic pain	*Tnfaip1*	Zhao et al. (2018)
47	rno-miR-200b-3p	Spinal cord and microglial cells of Sprague-Dawley rats with CCI-induced neuropathic pain	*Zeb1*	Yan et al. (2018b)
48	rno-miR-429			
49	rno-miR-544-3p	Microglial cells of Sprague-Dawley rats with CCI-induced neuropathic pain	*Stat3*	Jin et al. (2018)
50	rno-miR-455-3p	Thalamus and anterior cingulate of Sprague-Dawley rats with complete brachial plexus avulsion-induced neuropathic pain	*Camk2b* and *Prkcg*	Liu et al. (2017b)
51	rno-miR-208a-3p			
52	rno-miR-32-3p			
53	rno-miR-146a-5p	Macrophages of Sprague-Dawley rats with streptozocin-induced diabetic peripheral neuropathy	*Traf6*	Ren et al. (2021)
54	rno-miR-28-5p	Spinal cord of Sprague-Dawley rats with CCI-induced neuropathic pain	*Zeb1*	Bao et al. (2018)
55	rno-miR-26a-5p	Spinal cord tissue of Sprague-Dawley rats with CCI-induced neuropathic pain	*Mapk6*	Zhang et al. (2018b)
56	rno-miR-381-3p	Dorsal spinal cord of Sprague-Dawley rats with CCI-induced neuropathic pain	*Hmgb1, Cxcr4*	Zhan et al. (2018)
57	rno-miR-134-5p	Sciatic nerve of Sprague-Dawley rats with CCI-induced neuropathic pain	*Twist1*	Ji et al. (2019)
58	rno-miR-136-5p	Dorsal spinal cord of rats with CCI-induced neuropathic pain	*Il6r*	Zhang et al. (2019a)
59	rno-miR-182	Dorsal root ganglion of Sprague-Dawley rats with spared nerve injury-induced neuropathic pain	*Scn9a*	Cai et al. (2018)
60	rno-miR-202-5p	Spinal dorsal horn of Sprague-Dawley rats with bilateral CCI-induced neuropathic pain	*Rap1a*	Fang et al. (2019)
61	rno-miR-98-5p	Dorsal spinal cord of Sprague-Dawley rats with CCI-induced neuropathic pain	*Stat3*	Zhong et al. (2019)
62	rno-miR-146b-5p	Sciatic nerve of rats with diabetic peripheral neuropathy	—	Luo et al. (2019)
63	rno-miR-340-5p	Spinal cord tissue and microglial cells of Sprague-Dawley rats with CCI-induced neuropathic pain	*Rap1a*	Gao et al. (2019)
64	rno-miR-30b-5p	Dorsal root ganglion of Sprague-Dawley rats with Oxaliplatin-induced peripheral neuropathic pain	*Scn8a*	Li et al. (2019a)
65	rno-miR-362-3p	Spinal cord of Sprague-Dawley rats with spinal cord injury-induced neuropathic pain	*Pax2*	Hu Y et al. (2019)
66	rno-miR-34a-5p	Dorsal root ganglion of Sprague-Dawley rats with CCI-induced neuropathic pain	Scn2b and Vamp2	Brandenburger et al. (2019)
67	rno-miR-20b-5p	Spinal dorsal horn and isolated microglia of Sprague-Dawley rats with CCI-induced neuropathic pain	*Akt3*	You et al. (2019)
68	rno-miR-129-5p	Lumbar spinal dorsal horn of Sprague-Dawley rats with bilateral CCI-induced neuropathic pain		Tian et al. (2020)
69	rno-miR-101a-3p	Lumbar spinal dorsal horn of Sprague-Dawley rats with CCI-induced neuropathic pain	Mtor	Xie et al. (2020)
70	rno-miR-1224	Dorsal root ganglion of Sprague-Dawley rats with spinal nerve injury-induced neuropathic pain	—	Dai et al. (2019)
71	rno-miR-488-3p			
72	rno-miR-1249			
73	rno-miR-212-3p	CCI-induced neuropathic pain in rats	*Scn3a*	Li et al. (2019c)
74	rno-miR-15a	Spinal cord tissue of Sprague-Dawley rats with peripheral nerve injury-induced neuropathic pain	*Akt3*	Cai et al. (2020b)
75	rno-miR-154-5p	Spinal cord tissue and microglia of Sprague-Dawley rats with CCI-induced neuropathic pain	*Aqp9*	Wu et al. (2020a)
76	rno-miR-672-5p	Dorsal spinal cord of Sprague-Dawley rats with CCI-induced neuropathic pain	—	Cao et al. (2019a)
77	rno-miR-542-5p			
78	rno-let-7d-5p			
79	rno-miR-342-5p			
80	rno-miR-675-5p			
81	rno-miR-329-5p			
82	rno-miR-194-5p	Sciatic nerve of Sprague-Dawley rats with CCI-induced neuropathic pain	*Foxa1*	Zhang et al. (2020a)
83	rno-miR-384-5p	Spinal cord tissue and dorsal root ganglion of Sprague-Dawley rats with CCI-induced neuropathic pain	*Scn3a*	Ye et al. (2020)
84	rno-miR-423-5p	Dorsal spinal cord of Sprague-Dawley rats with spinal nerve ligation-induced neuropathic pain	—	Pan et al. (2020)
85	rno-miR-547-5p	Spinal dorsal horn and dorsal root ganglion of Sprague-Dawley rats with CCI-induced neuropathic pain	*Il33, St2*	Zhou et al. (2020)
86	rno-miR-503-5p	Schwann cells of Sprague-Dawley rats with streptozocin-induced diabetic peripheral neuropathy	—	Wang et al. (2020a)
87	rno-miR-223-5p			
88	rno-miR-483-3p			
89	rno-miR-483-5p			
90	rno-miR-673-3p			
91	rno-miR-125b-5p	Sciatic nerve of Sprague-Dawley rats with streptozocin-induced diabetic neuropathy	—	Sun et al. (2020)
92	rno-miR-24-1-5p	Prelimbic cortex of Sprague-Dawley rats with spared nerve injury-induced neuropathic pain	—	Cai et al. (2020a)
93	rno-let-7i-3p	Sciatic nerve of Sprague-Dawley rats with streptozocin-induced diabetic neuropathy	—	Li et al. (2020c)
94	rno-miR-106b-5p			
95	rno-miR-107-3p			
96	rno-miR-1188-3p			
97	rno-miR-1193-3p			
98	rno-miR-140-3p			
99	rno-miR-181a-5p			
100	rno-miR-181b-2-3p			
101	rno-miR-1949			
102	rno-miR-211-3p			
103	rno-miR-214-5p			
104	rno-miR-219b			
105	rno-miR-23a-3p			
106	rno-miR-24-2-5p			
107	rno-miR-25-5p			
108	rno-miR-299a-5p			
109	rno-miR-3074			
110	rno-miR-324-5p			
111	rno-miR-325-5p			
112	rno-miR-326-5p			
113	rno-miR-329-3p			
114	rno-miR-335			
115	rno-miR-345-5p			
116	rno-miR-3551-5p			
117	rno-miR-3573-3p			
118	rno-miR-3594-3p			
119	rno-miR-369-3p			
120	rno-miR-379-5p			
121	rno-miR-497-5p			
122	rno-miR-500-3p			
123	rno-miR-500-5p			
124	rno-miR-532-5p			
125	rno-miR-551b-5p			
126	rno-miR-6216			
127	rno-miR-674-3p			
128	rno-miR-702-3p			
129	rno-miR-770-5p			
130	rno-miR-7b			
131	rno-miR-802-5p			
132	rno-miR-22-3p	Dorsal spinal cord tissues of Sprague-Dawley rats with CCI-induced neuropathic pain	*Eno1*	Li et al. (2020b)
133	rno-miR-216a-5p	Dorsal root ganglion of Sprague-Dawley rats with CCI-induced neuropathic pain	*Kdm3a*	Wang and Li (2020)
134	rno-miR-124-3p	Spinal dorsal horn of Sprague-Dawley rats with CCI-induced neuropathic pain	*Jag1*	Li et al. (2020a)
135	rno-miR-30a-3p	Microglial cells of Sprague-Dawley rats with CCI-induced neuropathic pain	*Ep300*	Tan et al. (2020)
136	rno-miR-133a-3p	Microglial cells of Sprague-Dawley rats with CCI-induced neuropathic pain	*Srpk1*	Li et al. (2020d)
137	rno-miR-186-5p	Spinal cord and astrocytes of rats with spinal cord injury-induced neuropathic pain	*Cxcl13*	Zhang et al. (2021b)
138	rno-miR-181b-5p	Microglial cells of rats with spinal nerve ligation-induced neuropathic pain	—	Liu et al. (2021)
139	rno-miR-138-5p	Rat lingual nerve tissue of Sprague-Dawley rats	—	Tavares-Ferreira et al. (2019)
140	rno-miR-141-5p	Dorsal root ganglion of rats with oxaliplatin-induced neuropathic pain	*Trpa1*	Zhang and Chen (2021)

## microRNAs as Diagnostic and Prognostic Markers in Neuropathic Pain

Huang et al. (2017) compared the levels of miRNA in the blood of postherpetic neuralgia (PHN) and acute herpes zoster (AHZ) patients. 157 serum miRNAs were differentially expressed in PHN patients than in AHZ patients. In comparison to AHZ patients, 17 serum miRNAs from PHN patients were overexpressed and 139 were underexpressed. According to the results of qRT-PCR, the levels of miR-892b, miR-127-5p, miR-107, miR-486-3p, and miR-34c-5p were all considerably greater in PHN patients than in AHZ patients. These miRNAs can be used as diagnostic markers to detect the progression of postherpetic neuralgia. Dayer et al. (2019) evaluated the expression changes of 184 circulating miRNAs in plasma samples from individuals with various origins of persistent pain. Following statistical analysis, 7 circulating miRNAs were discovered that were differentially expressed depending on whether the pain was nociceptive or neuropathic. Two circulating miRNA signatures (hsa-miR-320a and hsa-miR-98-5p) accurately classified the pain type of 70% of patients in the validation set. To summarize, circulating miRNAs are promising biomarkers for identifying and characterizing chronic pain types, as well as for improving the treatment of chronic pain. Peng et al. (2019b) explored miR-124, miR-183, and miR-338 as diagnostic biomarkers in a CiPN mice model. Among the three miRNAs that were analyzed, only miR-124 was statistically significantly increased. Cold allodynia and axonal degeneration were caused by high levels of circulating miR-124 in both the DRG and the sciatic nerve. Hence, plasma levels of miR-124 may be a good diagnostic biomarker for CiPN. Chu et al. (2019) investigated the role of miR-448 as a prognostic biomarker in neuropathic pain. miR-448 was consistently increased in CCI rats, while miR-448 downregulation reduced thermal hyperalgesia and mechanical allodynia in CCI rats. In CCI rats, the expression levels of IL1, IL6, and TNF were substantially higher, but these effects were reversed after treatment with a miR-448 inhibitor. miR-448 increased neuropathic pain in CCI rats via controlling neuroinflammation. Hence, upregulated miR-448 could be used for the prognosis of neuropathic pain.

## Regulatory Effects of miRNAs in Inflammation- and Diabetes-Associated Neuropathic Pain

### Regulatory Effects of miRNAs in Inflammation-Associated Neuropathic Pain

We discuss herein a few examples of specific miRNAs that play key regulatory roles in inflammation-associated neuropathic pain.

#### hsa-miR-101

Liu et al. (2019) studied the expression of miRNAs in plasma samples of patients with neuropathic pain and reported a significant downregulation of miR-101. KPNB1, an important regulator for NF-κB/p65 nuclear importing, was identified as a direct target of miR-101 in human embryonic kidney HEK293T cells. Thus, miR-101 inhibits NF-κB signaling via targeting KPNB1 resulting in downregulation of inflammatory cytokines IL-1β and TNF-α.

#### mmu-miR-128

Yang et al. (2017b) reported that miR-128 was downregulated in murine microglial BV2 cells (treated with spinal cord segment-derived conditioned medium of male Sprague-Dawley rats following spinal cord injury) and that overexpression of miR-128 altered the M1/M2 microglial gene expression. M1 phenotypic markers like CD32 and CD86 were significantly downregulated while M2 phenotypic markers like CD206 and Arg1 were upregulated. Moreover, inflammatory cytokines like IL-6, TNFβ and TNFα were markedly suppressed following upregulation of miR-128. Further, it was reported that phosphorylated p38 (phospho-p38) was downregulated after overexpression of miR-128 suggesting a key role for miR-28 in the pathogenesis of neuropathic pain.

#### mmu-miR-23a

In pathogen-free adult male C57BL/6J wild-type mice, Pan et al. (2018) studied the functional regulatory role of miR-23a in pain and its association with chemokine CXC receptor 4 (CXCR4) which has been implicated in neuropathic pain. It was observed that expression of CXCR4 was increased in murine spinal glial cells induced with neuropathic pain via partial sciatic nerve ligation (pSNL). miR-23a was observed to bind directly to CXCR4-3′UTR resulting in downregulation of spinal CXCR4. Finally, downregulation of miR-23a increased thioredoxin-interacting protein (TXNIP) which is linked with induction of NOD-like receptor protein 3 (NLRP3) inflammasome resulting in elevated pain behavior.

#### rno-miR-146a-5p

miR-146a-5p plays an important role in downregulation of IL-1 receptor (toll/interleukin-1 receptor; TIR) and toll-like receptor (TLR4) signaling pathways. TLR4 is activated in neuropathic pain as a key innate immune receptor (Li et al., 2014). TLR4 activation leads to production of proinflammatory cytokines like TNF-α and IL-6 due to nuclear translocation of NF-κB via activation of TRAF6 and IRAK1 (Allette et al., 2017). In a chronic constriction injury model using Sprague-Dawley rats, Wang et al. (2018) demonstrated that NF-κB-dependent miR-146a-5p suppressed IRAK1/TRAF6 which plays a major role in TIR signaling pathway. Further, miR-146a-5p alleviated neuropathic pain by suppressing IRAK1 and TRAF6 via inhibition of TLR4/NF-κB signaling pathway.

#### rno-miR-34c-5p

miR-34c-5p was found to be involved in neuropathic pain via SIRT1 and STAT3 signaling pathway. In a chronic constriction injury model using male Sprague-Dawley rats, it was reported (Mo et al., 2020) that SIRT1 is suppressed by miR-34c-5p resulting in activation of STAT3 signaling pathway. This promoted the release of inflammatory factors like TNF-α, IL-6 and IL-1β eventually inducing neuropathic pain.

We summarize in Table 7 the role of different miRNAs in neuropathic pain-associated inflammation.

**TABLE 7:** Role of different miRNAs in neuropathic pain-associated inflammation.

miRNA	Role of different miRNAs in neuropathic pain-associated inflammation	References
hsa-miR-103	Neuropathic chronic pain is alleviated by miR-103. Targets voltage-gated calcium channels (Cav2.1 and Cav2.2)	Favereaux et al. (2011)
hsa-miR-19b-3p	Higher levels observed in neuroinflammation and severe neuropathy. Positive association with pain seen when use of opioid is adjusted.	Ye et al. (2021)
hsa-miR-21	An anti-inflammatory miRNA that effectively modulates neuroinflammation by targeting Smad7 (TGF-β signaling repressed) and Spry1 (MAPK signaling boosted)	Gaudet et al. (2018)
hsa-miR-146a	miR-146 negatively regulates inflammation and is induced by activation of NFκB. Also, inhibits mRNAs that translate IRAK1 and TRAF6	Gaudet et al. (2018)
mmu-miR-23a	Increases chemokine CXC receptor 4 (*Cxcr4*) activity by targeting TXNIP/NLRP3 inflammasome axis	Pan et al. (2018)
mmu-miR-142-3p	Targets high mobility group box 1 (*Hmgb1*) to relieve neuropathic pain	Zhang et al. (2017)
rno-miR-146a-5p	Suppresses IRAK1/TRAF6 signaling pathway and reduces neuropathic pain	Wang et al. (2018)
rno-miR-32-5p	Downregulates dual-specificity phosphatase 5 (*Dusp5*)	Yan et al. (2018a)
rno-miR-150	Reduces neuropathic pain by toll-like receptor 5 (TLR5) inhibition	Ji et al. (2018)
rno-miR-26a-5p	Suppresses neuroinflammation and neuropathic pain. MAPK6 is the direct target and its upregulation reverses effect of miRNA	Zhang et al. (2018b)
rno-miR-128-3p	An inflammation mediator, zinc finger E-box binding homeobox 1 (*Zeb1),* is the target whose upregulation leads to neuropathic pain promoting neuroinflammation	Zhang et al. (2020c)

### Regulatory Effects of miRNAs in Diabetes-Associated Neuropathic Pain

We discuss herein a few examples of specific miRNAs that play key regulatory roles in diabetes-associated neuropathic pain.

#### hsa-miR-199a-3p

In a study conducted by Li et al. (2017b), 2), miR-199a-3p was reported to be downregulated in plasma samples of diabetic patients as compared to healthy controls. miR-199a targeted SERPINE2 by binding to the 3′UTR of SERPINE2 and promoted coagulation resulting in the development of diabetic neuropathy. miR-199a-3p was also found to suppress tissue plasminogen activator (tPA) pathway via regulation of SERPINE2 expression which lies upstream of the tPA pathway.

#### mmu-miR-193a

Wu et al. (2019) reported downregulation of miR-193a to alleviate neuropathic pain in male Balb/c mice induced with diabetes by streptozotocin. miR-193a targeted high mobility group box protein 1 (HMGB1) by binding to the HMGB1 3′-UTR region. HMGB1 proteins are key proinflammatory mediators resulting in abnormal inflammation response. Upregulation of miR-193a showed a downregulation of inflammatory cytokines like IL-6, IL-1β, and TNF-α in the lumbar spinal dorsal horn of diabetic mice.

#### rno-miR-9

miR-9 was reported (Sun et al., 2020) to be highly upregulated in STZ-induced Sprague-Dawley rats. Insulin gene enhancer binding protein-1 (ISL1) was identified as a target of miR-9 which bound to 3′UTR of ISL1. ISL1 plays a key role in activation of insulin gene transcription of pancreatic beta-cells. Further, ISL1 modulated the sonic hedgehog (SHH) signaling pathway to improve diabetic peripheral neuropathy. miR-9 inhibited the expression of ISL1 as well as SHH signaling pathway resulting in development of diabetic peripheral neuropathy.

**FIGURE 7:**
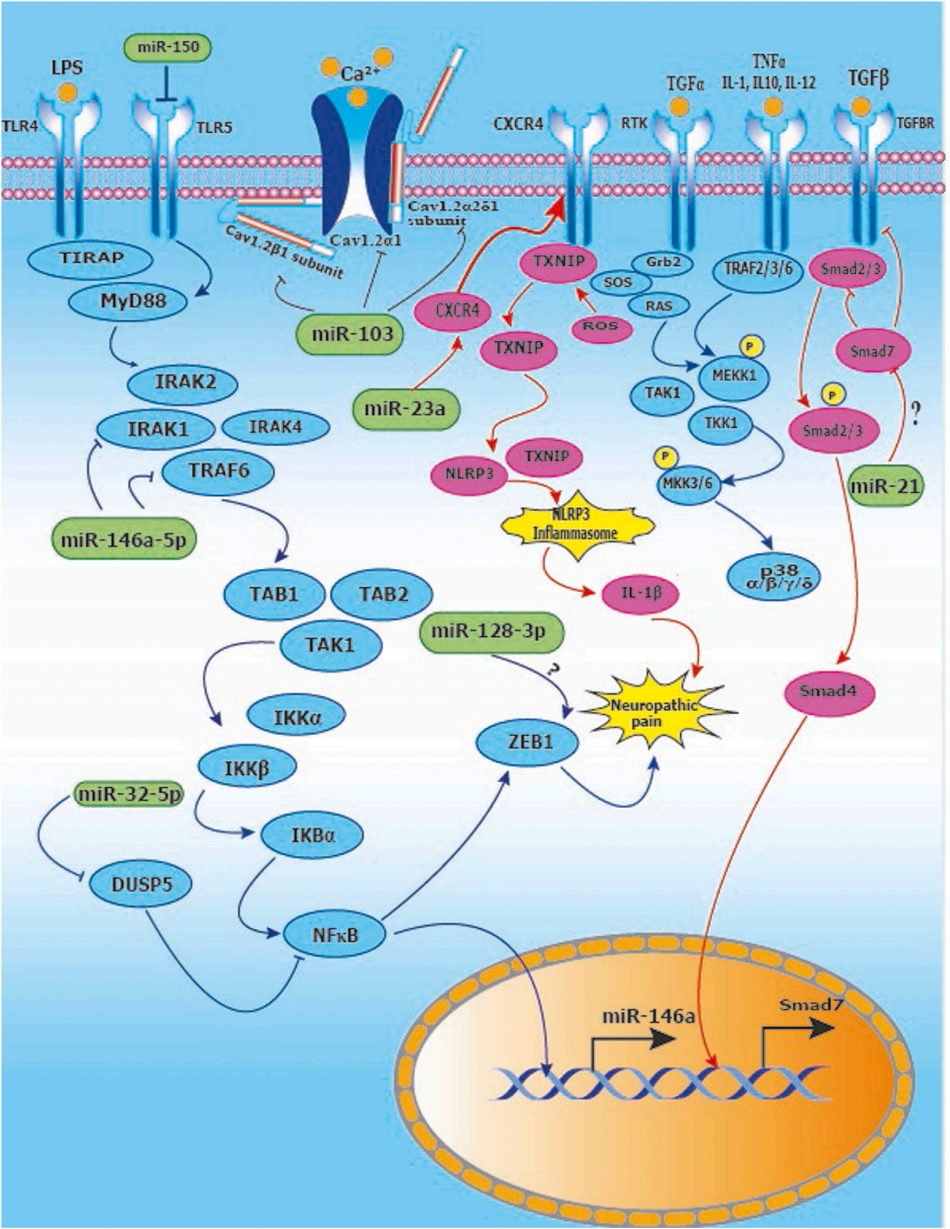
Signaling pathways modulated by miRNAs in neuropathic pain: **(A)** Activation of TLR4 and downstream IRAK/TRAF6 signaling leads to upregulation of NFĸB and ZEB1 leading to neuropathic pain. miR-146a-5p inhibits IRAK1 and TRAF6 which are upstream of NFĸB. Further, miR-32-5p inhibits DUSP5 resulting in suppression of NFkB signaling. miR-128-3p interacts with ZEB1, however, the nature of the interaction remains to be explored; **(B)** Inducing miR-103 suppresses the expression of subunits Cav1.2-α1, Cav1.2-α2δ1 and Cav1.2-β1 of Cav1.2-comprising L-type calcium channel thus relieving neuropathic pain; **(C)** Suppression of miR-23a results in upregulation of CXCR4 which mediates neuropathic pain via the TXNIP/NLRP3 inflammasome axis; **(D)** The p38 MAP Kinase pathway is implicated in neuropathic pain and miR-26a-5p downregulates MKK3/6 upstream of p38, whereas miR-21 inhibits Spry1 leading to RAS inhibition in the MAP Kinase signaling pathway.

Since diabetes plays an important role in the etiopathogenesis of neuropathic pain, we summarize key microRNA changes in neuropathic pain in Table 8 including miRNAs of human, mouse and rat origin. Further, we summarize various signaling pathways modulated by miRNAs in neuropathic pain in Figure 7 for the benefit of the reader.

**TABLE 8:** microRNA changes in neuropathic pain based on diabetes.

miRNA	Biological matrix (cell line/animal model/patient)	Modulation	References
hsa-miR-199a-3p	Blood plasma	Upregulated	Li et al. (2017b)
hsa-miR-499a	Peripheral blood	Upregulated	Ciccacci et al. (2018)
hsa-miR-216a	Blood	Upregulated	Li et al. (2021b)
hsa-miR-377	Blood	Upregulated	Li et al. (2021b)
hsa-miR-34a	Hippocampal postmortem tissue	Upregulated	Santos-Bezerra et al. (2021)
hsa-miR-34b	Hippocampal postmortem tissue	Upregulated	Santos-Bezerra et al. (2021)
hsa-miR-34c	Hippocampal postmortem tissue	Upregulated	Santos-Bezerra et al. (2021)
hsa-miR-29a	Serum	Upregulated	Sun et al. (2020)
hsa-miR-9	Serum	Upregulated	Sun et al. (2020)
hsa-miR-23a	Peripheral blood mononuclear cells	Upregulated	Amin et al. (2020)
hsa-miR-23b	Peripheral blood mononuclear cells	Upregulated	Amin et al. (2020)
hsa-miR-23c	Peripheral blood mononuclear cells	Upregulated	Amin et al. (2020)
mmu-miR-210-3p	Lumbar spinal dorsal horn	Upregulated	Gong et al. (2015)
mmu-miR-98-5p	Lumbar spinal dorsal horn	Upregulated	Gong et al. (2015)
mmu-miR-34c	Trigeminal ganglion tissue	Upregulated	Hu J et al. (2019)
mmu-miR-341	Dorsal root ganglion	Upregulated	Cheng et al. (2015)
rno-miR-9	Sciatic nerves	Upregulated	Sun et al. (2020)
mmu-miR-27a	Schwann cell exosomes	Downregulated	Wang et al. (2020b)
mmu-miR-193a	Lumbar spinal dorsal horn	Downregulated	Wu et al. (2019)
mmu-miR-106a	Dorsal root ganglion	Downregulated	Wu et al. (2017)
rno-miR-146b-5p	Sciatic nerves	Downregulated	Luo et al. (2019)

## miRNAs and Epigenetic Mechanisms of Neuropathic Pain

Epigenetic events such as covalent histone modifications and DNA methylation regulate gene expression (Liang et al., 2015). Enzymes involved in these processes are histone acetyltransferase (HAT) and histone deacetylases (HDACs) for histone modification; and DNA methyltransferases (DNMTs) and demethylation enzymes (translocation dioxygenases) for DNA methylation. A study reported that CpG islands present in promoter region of miR-129 were hypermethylated by complete Freund’s adjuvant (CFA). This process of methylation modulated chronic inflammatory pain by targeting Ca2+/calmodulin-dependent protein kinase γ (CaMKIIγ) (Liang et al., 2015) and (Pan et al., 2014). Given the epigenetic mechanisms involved in neuropathic pain, there is potential for therapeutic intervention by targeting miRNAs and/or their targets. The epigenetic alterations mediated by miRNAs result in either degradation of target mRNAs or translational repression. Epigenetic mutations are not reversed when they are persistent for a long period without any intervention, this is termed as “metabolic memory” of the target cell wherein the epigenetic alterations occurred (Yamunadevi et al., 2021). Further, Liu et al. (2020) reported an isoform of DNA methyltransferase (DNMT3a) that hypermethylates the promoter region of miR-214-3p. This led to the inhibition of expression of miR-214-3p in rats with spinal nerve ligation. Zebularine, a DNMT inhibitor, abrogated the suppression of miR-214-3p expression, resulting in the reduction of cytosine methylation in the promoter region; therefore, decrease in colony-stimulating factor-1 (CSF1) was observed as miR-214-3p negatively regulated expression of CSF1. As CSF1 decreased, neuropathic pain decreased subsequently. Thus, neuroinflammation and neuropathic pain were induced by increased production of CSF1 as miR-214-3p was epigenetically suppressed by DNMT3a (Liu et al., 2020). Furthermore, Tan et al. (2020) studied the role of miR-30a-3p in sciatic nerve CCI Sprague Dawley rats. rno-miR-30a-3p targeted E-cadherin transcriptional activator (EP300) which further upregulated brain-derived neurotrophic factor (BDNF); this resulted in increased neuropathic pain as EP300 and BDNF both were directly involved in neuropathic pain (Tan et al., 2020).

Kcna2, a voltage-dependent potassium channel mRNA, is inhibited by a conserved lncRNA, Kcna2 antisense RNA. This results in the decreased expression of Kcna2 channel. The decrease in voltage-dependent potassium channel resulted in alleviation of neuropathic pain and increase in excitability (Zhao et al., 2013). Further, methyl-CpG-binding domain protein (MBD1) is an epigenetic repressor that modulates gene transcription. Using MBD1-deficient (Mbd1^−/−^) mice with spinal nerve ligation, it was demonstrated that MBD1 recruited DNMT into the gene promoters of Kcna2 and Oprm1 gene and repressed their expression. Hence, regulation of DNMT-controlled expression of Kcna2 gene in dorsal root ganglion neurons led to neuropathic pain (Mo et al., 2018). Interestingly, Zhang et al. (2021a) reported the key role of voltage-gated potassium channels (Kv) in regulation of neuropathic pain induced by nerve injury. In dorsal root ganglion and spinal cord of naïve and CCI Sprague Dawley rats, neuron excitability and Kv currents were examined which showed that the downregulation of Kv1.2 induced hypersensitivity in naïve rats. As Kv1.2 was downregulated, the expression of miR-137 was increased which targeted Kcna2 and regulated it. Hence, by inhibiting miR-137, the Kv1.2 expression was upregulated restoring excitability and abnormal currents. As a result, when voltage-gated potassium channels (Kv1.2) was restored, it contributed to alleviation of neuropathic pain proving to be a novel therapeutic target (Zhang J. et al., 2021).

## Conclusion and Future Perspectives

Complexity, progressive nature, and improper identification of neuropathic pain make it difficult to manage. Several clinical studies have been carried out in recent years in order to alleviate the poor quality of life associated with neuropathic pain using tricyclic antidepressants, opioid analgesics, physical and psychological therapies. However, these treatments are not sufficient for the management of neuropathic pain. Given the improper diagnosis of neuropathic pain, efforts have been made to identify miRNAs that can serve as biomarkers of neuropathic pain. Indeed, several miRNAs are modulated in the etiopathogenesis of neuropathic pain. It is, hence, important to study miRNA-miRNA and miRNA-gene target networks and evaluate the miRNA interactome in the preventative or therapeutic management of neuropathic pain. Moreover, it is clear that neuropathic pain is driven by neuroinflammation and nerve damage. Various miRNAs regulated in neuroinflammation and nerve damage may be especially useful as biomarkers for diagnosis and as therapeutic targets for the management of neuropathic pain. Taken together, experimental research aimed at deepening our knowledge of the miRNA interactome will be necessary in the near future to evaluate these exciting candidate biomarkers in the management of neuropathic pain.

## Abbreviations

Gla, gamma-carboxyglutamate; GAS6, growth arrest-specific 6; DN, diabetic neuropathy; PN, peripheral neuropathy; DPN, diabetic peripheral neuropathy; T2DM, type 2 diabetes mellitus; RANKL/RANK, receptor-activator for nuclear factor kappa B ligand; TLDA, TaqMan low density array; CCI, chronic constriction injury; DRG, dorsal root ganglion; PHN, post-herpetic neuralgia; AHZ, acute herpes zoster; hsa, *Homo sapiens*; mmu, *Mus musculus*; rno, *Rattus norvegicus*.
